# Racism as Public Health Crisis: Assessment and Review of Municipal Declarations and Resolutions Across the United States

**DOI:** 10.3389/fpubh.2021.686807

**Published:** 2021-08-11

**Authors:** Dara D. Mendez, Jewel Scott, Linda Adodoadji, Christina Toval, Monica McNeil, Mahima Sindhu

**Affiliations:** ^1^Department of Epidemiology, Graduate School of Public Health, University of Pittsburgh, Pittsburgh, PA, United States; ^2^Department of Psychiatry, School of Medicine, University of Pittsburgh, Pittsburgh, PA, United States; ^3^Department of Behavioral and Community Health Sciences, Graduate School of Public Health, University of Pittsburgh, Pittsburgh, PA, United States; ^4^School of Health and Rehabilitation Sciences, University of Pittsburgh, Pittsburgh, PA, United States; ^5^Departments of Communication and Statistics, Dietrich School of Arts and Sciences, University of Pittsburgh, Pittsburgh, PA, United States

**Keywords:** racism, public health, health equity, declarations, legislation, policy

## Abstract

Racism in the United States has been cited as a key driver of racial health inequities. Racism as a public health crisis has been in the forefront, particularly with respect to state and municipal governments that have developed legislation, resolutions, and declarations. This policy brief includes a review of resolutions and declarations across the US related to Racism as a Public Health Crisis through the end of September 2020. There were 125 resolutions reviewed for content related to the history of racism, reference to racial health equity data, content related to action steps or implementation, and any accompanying funding or resources. We found that the majority of policies name racism as critical in addressing racial inequities in health with limited details about specific actions, funding, or resources.

## Introduction

Racial inequities in health and well-being are well-documented. For example, there are long-standing racial inequities in maternal and infant health (e.g., maternal mortality and preterm birth), chronic disease (e.g., diabetes and hypertension), and COVID-19 cases, hospitalizations, and deaths (1–6). These racial inequities are a result of historical and contemporary oppression. Recognizing and naming racism as a public health crisis is a critical first step in dismantling structures and systems of oppressions that not only impede health and well-being but contribute to racial inequities in health. Naming racism as a public health crisis also has implications for other systems that are important for health, such as education, food systems, housing, and employment.

Racism can be defined as “state-sanction and/or extralegal (meaning not necessarily regulated by the law) production and exploitation of group-differentiated vulnerability to premature death” (7) and elevate how oppression, privilege, and power operate based on racial classifications (8). The origin of the United States includes colonization, genocide, and land theft from Indigenous communities as a result of white supremacy and structural racism. The trauma and oppression committed against Indigenous communities has implications for health and well-being (8). Dr. Camara Jones, prominent public health expert and former president of the American Public Health Association, defines racism as “a system of structuring opportunity and assigning value based on the social interpretation of race, that unfairly disadvantages some individuals and communities, unfairly advantages other individuals and communities, and saps the strength of the whole society through the waste of human resources (9).” Racism also can operate at three levels, as institutionalized, interpersonal, or personally-mediated and then internalized (10), with structural being the totality of ways in which racism operates in a society.

The majority of empirical research on racism and health has focused on interpersonal or personally-mediated racism (which includes the day-to-day experiences of racism that are mediated through individuals and a result of racist structures and institutions). This research demonstrates that personally-mediated racism acts through stress pathways to dysregulate bodily systems, resulting in accelerated aging and adverse health outcomes; and daily stressors accumulate as “wear and tear on the body” also known as “weathering (11, 12).” As a result, immune function is dysregulated and the body cannot reach equilibrium, creating vulnerability and susceptibility to disease, which challenges the body's ability to remain in a healthy state (11, 12).

There is also empirical evidence describing how structural and institutional racism shape health and well-being. A large body of this research measures residential segregation as a fundamental cause of health inequity (13). Segregation is a result of specific policies and actions that disproportionately affect communities of color, particularly Black communities, and low-income communities. Racist practices, policies and structures have resulted in disparate social and physical environmental conditions, limiting access to healthcare resources, inequities in housing and wealth attainment, influencing behaviors, and ultimately health (14). Residential segregation is the result of housing policies such as redlining, backed by the federal government. Broadly defined, racial redlining encompasses not only the direct refusal to lend in neighborhoods of color and Black neighborhoods in particular, but also procedures that discourage the submission of mortgage loan applications from these areas, and marketing policies that exclude such areas (15–17). Prior research highlights the intersections between lending disparities and health disparities (18–20). There is a growing body of research measuring structural racism in the form of racial inequities in political participation, judicial treatment, and employment and job status and its association with adverse health outcomes such as infant mortality, small for gestational age birth, cancer, and myocardial infarction (21–25). This work highlights how policies, structures, power, and privilege are fundamental in understanding and eliminating health inequities.

Policy and legislation specifically related to racism and health has come to the forefront over the past few years. Examples include federal policy such as the Anti-Racism in Public Health Act of 2020, the Black Maternal Momnibus Act of 2020 (and Act of 2021) as well multiple local and state resolutions related to Racism as a Public Health Crisis (26, 27). In May 2019, Milwaukee County in Wisconsin was one of the first areas to develop and adopt a resolution related to racism as a public health crisis. Soon after, several other locations adopted legislation or resolutions such as the city of Milwaukee (July 2019); Cook County, Illinois (June 2019); and Pittsburgh, Pennsylvania (December 2019). By the Spring of 2020, numerous cities, counties, and other governmental agencies adopted similar resolutions or declarations. Many of these resolutions were adopted after the killing of Breonna Taylor and George Floyd by police, the global movement for Black lives and a time of racial reckoning in 2020. There is not a formal public health definition of crisis; however, public health emergencies and disasters point to major events affecting multiple populations causing trauma or harm (28). Racism as a public health crisis also speaks to trauma or harm over time and over multiple generations. Simultaneously, public health crises would require long-term solutions (29); solutions that address structures and institutions that create health inequities.

In this paper, we review declarations and resolutions adopted by cities, counties and states prior to October 2020 related to Racism as a Public Health Crisis. The purpose is to provide an analysis of the key content, how racial inequity in health and other areas are articulated in these resolutions and specific actions steps to name racism and ultimately eliminate racism. These resolutions specifically point to race as a social construct, racism and its relation to health, and variations in action items on how to undo or address racism. We hypothesize that many legislators and governing bodies have adopted resolutions that take the critical first step to naming racism as a public health crisis, but there will be limited language in the legislation that explicitly include action steps (e.g., programs, funding) to eliminate racism.

## Policy Overview and Methods

Policies, declarations and other legislation adopted by cities and municipalities in the United States were identified by searching publicly available search engines. Two team members independently searched public engines and databases (May–June 2020) while two additional team members (October–December 2020) updated the list while also reviewing state and city government websites and information generated from the American Public Health Association. Given the recency of most policies and declarations, and their dissemination in the lay literature and governmental websites, public search engines were the best option for identifying legislative documents. No limitation was set on the initiation date of the policy, and all resolutions and legislations passed prior to October 1, 2020 were reviewed.

The policies and resolutions were organized by state and reviewed by at least one researcher. Data were extracted into a structured, cloud-based, and data table. Co-authors met and discussed the content of the resolutions. Data initially recorded in the table included location, date approved, any background on the process for developing the resolution, the person or group who introduced the resolution, overall content, themes, and proposed actions. After data extraction of the first one-third of the resolutions, co-authors met and discussed common recurring themes, overlap across resolutions and a consolidated list of 18 themes. The data table was updated to record themes present in each legislation from the pre-identified list. As a result of the discussion, the following substantive areas were analyzed: historical context of racism, if maternal child health was addressed, if economic policies were addressed, funding described, names of any organizations, or entities mentioned.

The research team met to review the structured data table to identify, patterns, similarities and differences across the resolutions and legislation. The group also compiled a list of professional health organizations (e.g., American Public Health Association) that have published formal statements declaring racism as a public health crisis. Although the position statements by professional organizations are outside of the scope of this review, we acknowledge examples in the discussion of this paper.

The aforementioned search of government (e.g., state, city, and town) policies and resolutions was complemented with an *ad hoc* search of PubMed of texts related to racism as a public health crisis. The purpose of the search was not to conduct a separate review of published literature on racism and health but to provide some potential context for the emergent policies. Search terms were “racism,” “public health,” and “crisis” combined with Boolean connectors. The search returned 60 texts that were reviewed for key content; none of which were specifically related to policies or resolutions on racism as a public health crisis and therefore not included in this review.

## Policy Options: Review of Resolutions and Declarations of Racism as Public Health Crisis

**Figure 1:**
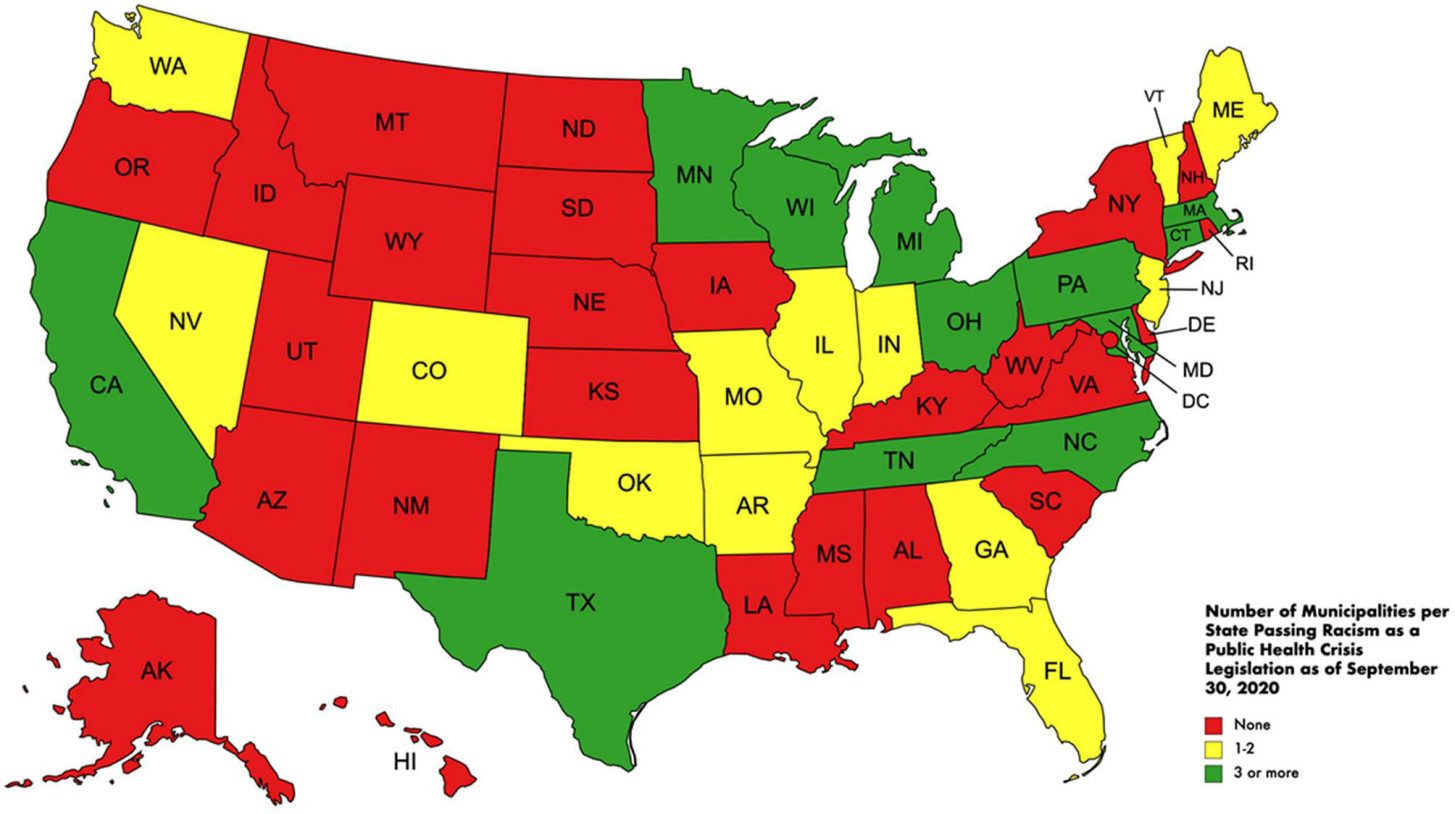
Geographical distribution of racism as a Public Health Crisis legislation through September 30, 2020.

As of October 1, 2020, there were 128 resolutions, declarations or legislative content related to racism as a public health crisis passed by state and local governments and municipalities (Table 1). There were declarations in a total of 25 states. By this time, only Wisconsin, Nevada and Michigan had a resolution passed at the state level. California, Massachusetts, Michigan, and Ohio had more than 10 municipalities/cities with resolutions or declarations. A total of 13 of the 25 states had fewer than three local municipalities/cities with resolutions (Table 1 and Figure 1). Additional local municipalities and states passed declarations or other legislative content after October 1, 2020. However, the research team chose this date to have sufficient time for review, analysis and write up of existing legislation up to that point; because we reached content saturation judged by minimal new findings; and repeated language that was “cut and paste” across multiple declarations. Additional declarations, resolutions, and legislations were passed in municipalities in Hawaii, Kentucky, Missouri, Utah, Virginia, Washington, DC, and West Virginia through the end April 2021.

**Table 1:** Racism as a Public Health Crisis legislation summary by state through September 30, 2020.

**State**	**Municipalities identifying racism** ** as a PH crisis** **prior to 10/1/20**	**Municipalities providing historical context of racism specific to the city,** **county, or state**	**Municipalities including maternal child health in the legislation on racism** **as a PH crisis**	**Municipalities including information on economic policies (e.g., living wage) in the legislation on racism** **as a PH crisis**	**Municipalities including plans for funding initiatives related to racism as a PH crisis**
Alabama	None	None	None	None	None
Alaska	None	None	None	None	None
Arizona	None	None	None	None	None
Arkansas	Fayetteville	None	Fayetteville	None	Fayetteville
California	Coachella, Fontana, Goleta, Indio, Los Angeles, Long Beach, Moreno Valley, Oxnard, Palm Springs, Redlands, Rialto, Riverside City, Riverside County, San Bernardino County, San Luis Obispo, Santa Barbara, Santa Clara County, Santa Cruz County, Ventura City, Yolo County	Goleta, Los Aneles, San Bernardino County, Santa Barbara	Los Angeles, Long Beach, Oxnard, Rialto, San Bernardino County, Santa Clara County, Santa Cruz County	None	Long Beach, San Luis Obispo, Santa Clara County, Ventura City
Colorado	Denver	Denver	None	None	None
Connecticut	Bloomfield, Bridgeport, Colchester, Easton, Glastonbury, Hamden, Hartford, Manchester, New Britain, New Haven, New London, Simsbury, South Windsor, West Hartford, Windham, Windsor	None	Bloomfield, Bridgeport, Hamden, New Britain, Simsbury, West Hartford, Windham, Windsor	None	None
Delaware	None	None	None	None	None
Florida	Hillsborough County	None	Hillsborough	None	None
Georgia	DeKalb County	None	DeKalb County	None	None
Hawaii	None	None	None	None	None
Idaho	None	None	None	None	None
Illinois	Cook County	None	Cook County	None	None
Indiana	Evansville, Indianapolis Marion County	Indianapolis Marion County	Indianapolis Marion County	Indianapolis Marion County	None
Iowa	None	None	None	None	None
Kansas	None	None	None	None	None
Kentucky	None	None	None	None	None
Louisiana	None	None	None	None	None
Maine	Portland	Portland	None	None	None
Maryland	Anne Arundel County, Montgomery County, Prince George County	Montgomery County, Prince George County	Montgomery County	None	None
Massachusetts	Beverly, Boston, Chicopee, Everette, Framingham, Holyoke, Longmeadow, Medford, Revere, Somerville, Springfield	None	Springfield	None	Beverly, Boston, Everette, Revere, Somerville
Michigan	Eaton County, Flint, Genesee County, Ingham County, Jackson, Kalamazoo County, Lansing, Pontiac, Port Huron, Washtenaw County, Wayne County, Westland, Ypsilanti, State At Large	Eaton County, Flint, Ingham County, Kalamazoo County, Lansing, Washtenaw County, State At Large	Eaton County, Genesee County, Ingham County, Kalamazoo County, Lansing, Pontiac, Washtenaw County, Westland, State At Large	None	Genesee County, Lansing, State At Large, Washtenaw County
Minnesota	Hennepin County, Minneapolis, Olmsted County, State At Large	Minneapolis	Minneapolis	None	Hennepin County, Minneapolis
Mississippi	None	None	None	None	None
Missouri	Kansas City	None	None	None	None
Montana	None	None	None	None	None
Nebraska	None	None	None	None	None
Nevada	State At Large	None	None	None	None
New Hampshire	None	None	None	None	None
New Jersey	Leonia	None	None	None	None
New Mexico	None	None	None	None	None
New York	None	None	None	None	None
North Carolina	Ashville, Charlotte, Durham, Mecklenburg County, New Hanover County, Pitt County, Wake County	Charlotte, Mecklenburg County	Ashville, Durham, Mecklenburg County, New Hanover County, Pitt County	Wake County	None
North Dakota	None	None	None	None	None
Ohio	Akron, Athen, Canton, Cincinnati, Cleveland, Columbus, Cuyahoga County, Dayton, Elyria, Franklin County, Hamilton County, Lima, Lorain County, Montgomery County, Piqua, South Euclid, Summit County, Stow, Upper Arlington, Westerville, Warren, Youngstown	Akron, Athen, Canton, Cincinnati, Cleveland, Columbus, Cuyahoga County, Dayton, Franklin County, Hamilton County, Montgomery County, Piqua, Summit County, Stow, Westerville, Warren	Akron, Athens	Cleveland	Columbus, Cuyahoga County
Oklahoma	Ardmore	None	None	None	None
Oregon	None	None	None	None	None
Pennsylvania	Allegheny County, Erie, Pittsburgh	Allegheny County, Erie, Pittsburgh	Allegheny County, Erie, Pittsburgh	None	Pittsburgh
Rhode Island	None	None	None	None	None
South Carolina	None	None	None	None	None
South Dakota	None	None	None	None	None
Tennessee	Chattanooga, Memphis, Shelby County	Memphis	Chattanooga	None	Chattanooga
Texas	Austin, Dallas County, Harris County, San Antonio	Austin, Dallas County, Harris County, San Antonio	Austin, Dallas County, Harris County, San Antonio	None	None
Utah	None	None	None	None	None
Vermont	Burlington	None	None	None	None
Virginia	None	None	None	None	None
Washington	King County	King County	King County	None	None
West Virginia	None	None	None	None	None
Wisconsin	Cudahy, Dane County, Kenosha County, Milwaukee, Milwaukee County, Rock County, State At Large	None	Cudahy, Dane County, Kenosha County, Milwaukee, Milwaukee County	None	Cudahy, Milwaukee
Wyoming	None	None	None	None	None

In the review of the resolutions and legislative content, the historical context of racism in the United States is usually discussed in the context of the nation's history of slavery and racist policies such as redlining. Almost half of the municipalities (45%) included examples of racism's historical context at a national level (Table 1). However, specific examples of racism at the local and state levels were slightly less common, with 30 and 14% of resolutions, respectively. The examples provided include Santa Barbara County, California, which named the first enslaved resident, John Forney, highlighting that Black people were enslaved outside of the southern U.S. Another example was Prince Georges County, Maryland which acknowledged that schools' desegregation in that county did not occur until 1972, 18 years after Brown vs. Board of Education declared school segregation unconstitutional. Westerville, Ohio, touted its long history of support of the abolitionist movement evidenced by several underground railroad stops and home to abolitionist Benjamin Hanby and Otterbein University, one of the first predominantly White institutions to accept men and women of color. Minneapolis explicitly acknowledges the placement of the city on Indigenous land. The three-page resolution of Harris County, Texas, was among the longest, and provided extensive detail on how racism was within the fabric of every institution and facet of life, in which they note that the County Attorney's office was a slave auction site and the Texas Rangers' murder and oppression of Black and Indigenous communities. These concrete examples of how historical contexts set the stage for current inequities were common across multiple resolutions (Supplementary Table 1A includes a detailed table with weblinks to the full resolution or legislative content).

The four most common themes in order of prevalence were systemic racism, COVID-19, social determinants of health (SDOH), and specific health outcomes. Many resolutions and declarations defined three levels of racism in the opening paragraphs as defined by Jones (10) and established the connection between health inequities and racism. Mentions of systemic racism were present in 92 of the 128 resolutions compared to 45 and 5 resolutions for interpersonal racism and hate crimes, respectively. For example, the template provided by Health Equity Solutions, a community-based organization in Connecticut, has language built in defining systemic racism (30). Several resolutions had shared language that was “cut and pasted” and applied across multiple municipalities and states, including language related to systemic racism and racism in general. Some of this copy and paste language included reference to action plans, definitions of racism and racial justice, and “400 Years of Black America's experience under slavery and Jim Crow” and related laws (Supplementary Table 1B).

Given that only five of the resolutions were adopted prior to 2020, it is not surprising that COVID-19 was the second most common theme (76 out of 128). Over half of the declarations included statistics or broad statements on the disproportionate impact of the COVID-19 pandemic on Black, Indigenous and Latinx communities. It seems that the confluence of COVID-19 and the death of George Floyd catalyzed a rapid proliferation of declarations of racism as a public health crisis as many of the declarations specifically note this. While COVID-19 was a common theme, police violence (28 of 128) and police reform (13 of 128) were less common components of the declarations (results not shown). There were also very few declarations that discussed economic inequities and related policies (Table 1 besides general statements about social determinants of health).

Multiple resolutions included content related to social determinants of health (SDOH) and specific health outcomes. A total of 66 resolutions discussed SDOH and 63 discussed health outcomes. Common language related to social determinants of health included naming key areas such as “housing, education, employment, transportation, and criminal justice (31).” Several resolutions included specific language related to maternal and child health (Table 1). Specific examples include racial inequities in infant and maternal mortality, key indicators used to capture the health of a nation.

In addition to background, definitions of racism, examples of health inequities, and overall rationale for the declarations and resolutions, we reviewed action items (Table 2) and whether specific funding was named or allotted (Table 1). Overall, action steps were frequently absent from the declarations of racism as a public health crisis. We classified action items as passive or active. Passive action steps convey a commitment to advocate for equitable policies and facilitate discussions on the impacts of systemic racism but fail to mention how municipalities explicitly plan to make equitable decisions moving forward. Various municipalities in nine states had mostly passive action steps. For example, Ardmore, Oklahoma, noted a need to consider tools to eliminate racism, but there is no indication of how they plan to achieve this. Municipalities in 16 states had overall active action steps. Active action steps include specific plans to enact equitable and systemic changes in neighborhoods and school districts as well as community investigations and future investment. For example, Ventura City, California, has committed to enacting police reform. They specifically aim to formalize a ban on excessive force and reiterate police department policies on these matters. Overall, action steps emphasized data-driven review, community reparations, police reform, and the creation of offices and committees geared toward racial equity.

**Table 2:** Summary of legislation implemented active and passive action steps related to racism as public health crisis.

**State**	**Examples of active action steps** **Include name of municipality**	**Examples of passive action steps** **Include name of municipality**	**Overall, are actions in the state more active or passive?**
Alabama	None	None	None
Alaska	None	None	None
Arizona	None	None	None
Arkansas	Foster small business development, affordable housing and community infrastructure serving minority residents and those of lower income (Fayetteville)	None	Active
	Develop and implement a Racial Equity Strategic Action Plan (Fayetteville)		
California	Commit to formalizing a ban on the use of excessive force and reiterating police departments policies about excessive force (Ventura)	Commitment to action that recognizes and addresses racism by ensuring meaningful progress (Yolo County)	Active
	Creation of an *ad hoc* Special Committee on Equity and Social Justice (Coachella)		
Colorado	The expansion of documented equity decision-making frameworks that are transparent to the public (Denver)	[We support]. agency organizational workplans to address and correct embedded policies that discriminate and perpetuate racism (Denver)	Passive
Connecticut	Improve the quality of the data collected and analyze using qualitative and quantitative data (New Britain)	Identify activities to enhance diversity and ensure antiracism principles (Easton)	Active
	Appoint a Commission on Racial Justice and Equity, composed of nine members (Glastonbury)		
Delaware	None	None	None
Florida	Periodic reports to assess progress and capitalize on opportunities to further advance racial equity (Hillsborough County)	Promote equity through all policies (Hillsborough County)	Passive
Georgia	Periodic reports to assess progress and capitalize on opportunities to further advance racial equity (DeKalb County)	Promote equity through all policies (DeKalb County)	Passive
Hawaii	None	None	None
Idaho	None	None	None
Illinois	Create the Office of Health and Social Equity (Cook County)	Encourage others [local, state and national entities to recognize racism as a public health crisis (Cook County)]	Active
Indiana	Investigating in disadvantaged neighborhoods that suffer most from racial disparities (Evansville)	Honest and open debate, discussion and analysis (Evansville and Indianapolis Marion County)	Active
Iowa	None	None	None
Kansas	None	None	None
Kentucky	None	None	None
Louisiana	None	None	None
Maine	Establish a Racial Equity Steering Committee (Portland)	None	Active
Maryland	Work with the county police department to implement police reform such as banning chokeholds and strangleholds (Anne Arundel and Prince George Counties)	Continue to advocate locally and nationally for relevant policies that improve health in communities of color (Montgomery County)	Active
Massachusetts	Development of a “Boston Health Equity Now” plan with details objective and measurable goals (Boston)	Promote racially equitable policies and community support (Springfield)	Active
	Compile specific race and ethnic data documenting health inequities (Beverly)		
	Establish Citizen Police Advisory Committee to the Mayor (Holyoke)		
Michigan	Increase the budget for the Public Health Department and Racial Equity Office (Washtenaw County)	Develop an anti-discrimination policy (Jackson County)	Active
Minnesota	Develop annual report with racially disaggregated data on the health of BIPOC and recommendations for actions to eliminate disparities and improve overall health (Minneapolis)	Study and investigate this issue with special emphasis on the services the county provides (Olmsted County)	Active
Mississippi	None	None	None
Missouri	Assess internal policies to ensure racial equity is a core element of the city (Kansas City)	Encourage other local, state, and national government to recognize racism as a public health crisis (Kansas City)	Passive
Montana	None	None	None
Nebraska	None	None	None
Nevada	None	Declare Racism a Public Health Crisis (State at Large)	Passive
New Hampshire	None	None	None
New Jersey	Encourage racial equity training among community partners, vendors contractors (Leonian)	Promote equity through policies (Leonian)	Passive
New Mexico	None	None	None
New York	None	None	None
North Carolina	Establish a Community Reparations Commission (Ashville)	Support policies that promote racial equity (Charlotte)	Passive
	Create periodic reviews to assess racial equity progress (Durham)	Encourage others (states, cities, counties) to promote racial equity policies (New Hanover County)	
North Dakota	None	None	None
Ohio	Systemic review of Canton City Public Health department programs (Canton)	Commits to open discussion on race and its impact (Lima)	Active
	Remove discriminatory laws (i.e. Stop and Identify Statute) (South Euclid)	Strengthen government/community partnerships (Lorain County)	
Oklahoma	None	Consider tools to eliminate racism (Ardmore)	Passive
Oregon	None	None	None
Pennsylvania	Create internal policies, procedures, and assessments for racial equity (Allegheny County, Pittsburgh)	Advocate for relevant policies to improve health of communities of color (Allegheny County, Pittsburgh)	Active
	Create status report to recommend and review policies to reduce racial disparities (Erie)	Commits to addressing racism (Erie)	
Rhode Island	None	None	None
South Carolina	None	None	None
South Dakota	None	None	None
Tennessee	Address minority health inequities, including a systematic, data-driven focus on poverty, economic mobility, inequities, and other factors that may impact the social determinants of health (Chattanooga)	Commitment to enacting policies that defend minorities and eradicate the effects of Systemic Racism (Shelby County)	Passive
Texas	Promoting racially equitable city services, programs and policies from neighborhood investment to infrastructure and transportation to economic and workforce development (San Antonio)	Develop policies, programs, and services that work to dismantle systemic racism (Harris County)	Active
Utah	None	None	None
Vermont	Develop a plan to ensure school district curriculum and teaching is culturally relevant and anti-racist (Burlington)	Actively fighting racist practices and participating in creating just and equitable systems (Burlington)	Active
Virginia	None	None	None
Washington	Board will assess, revise, and write guiding documents and policies with a racial justice and equity lens (King County)	Advance a public health approach in addressing institutional and systemic racism (King County)	Active
West Virginia	None	None	None
Wisconsin	Incorporate inclusion and equity in county practices, offer educational trainings/activities to employees and provide tools for members to engage actively with communities of color (Milwaukee County)	Advocate for relevant policies to improve health in communities of color (Kenosha County)	Active
Wyoming	None	None	None

Approximately 22 (17%) resolutions had explicit language related to funding for initiatives and actions (Table 1). Most of these funding plans were not concrete plans but rather an investment consideration, a vague suggestion of budget reallocation, or merely a recognition of the need to acquire funds somehow. In this regard, many of the funding initiatives could be described as passive. Only four municipalities/cities (Long Beach, San Luis Obispo, Boston, and Lansing) explicitly mention the amount of funding they plan to put toward their proposed action steps and reform. Long Beach's city budget for the 2020–21 fiscal year includes $3.2 million in funding for racial equity. San Luis Obispo plans to allocate $160,000 of city funds for diversity and inclusion. Boston plans to reallocate $3 million of its $414 Million police budget to these matters of public health. Lansing proposed $100,000 from Police Department Budget, $20,000 from Mayor's Office, and $50,000 from the Human Relations and Community Service office to put toward an Equity and Anti-Discrimination Fund.

## Discussion and Recommendations

In our review of municipal and state government resolutions and declarations related to Racism as a Public Health Crisis, more than 100 areas adopted resolutions, and there was a tremendous increase in resolutions in 2020 by the Spring and Summer. The adoption of these policies can be framed as a first step in naming racism as critical in addressing racial health inequities and other racial inequities in housing, transportation, and policing. Public Health Critical Race Praxis (PHCRP), which is an extension of Critical Race Theory to the field of public health, argues that racial inequities in health should not only be identified and named but eliminated (32). Given that over 100 declarations and resolutions garnered enough support to pass in multiple state and local governments is worthy of celebration, and simultaneously signals an opportunity to advocate for further action to eliminate racism. Also, the majority of the declarations and resolutions passed are led by local city, town and municipal governments. There is strength in having local entities lead local efforts, but there are some limitations when action and investment at the state and federal level do not follow.

PHCRP also acknowledges that understanding how racism shapes health is central for further action. A number of policies included language related to the levels of racism. The focus on systemic racism is meaningful because it uplifts the importance of large-scale solutions that are required to remedy the pervasive and malignant problem of racism. The focus on systemic racism in many of the resolutions and declarations may also be a result of the use of copy and paste templates that served to guide governing bodies in drafting the declarations. These declarations referenced definitions of racism, racial justice and historical and contemporary examples oppression, particularly experienced by Black communities in some of the “copy and paste” language. Although many of the declarations use general language to describe racism and its impact on multiple communities (e.g., Black, Indigenous, and Latinx), we identified the limited reference to historical oppression experienced by Indigenous populations and limited contemporary examples with the exception of the framing and definition of racism and the disproportionate impact of COVID-19 on Black, Indigenous, and Latinx communities.

There was some regional clustering of resolutions across several northern states and California. Some of the early states to adopt legislation before 2020 included the northern states such as Wisconsin, Michigan, and Pennsylvania. In reviewing, a source produced by the American Public Health Association that regularly updates its list of state and municipal resolutions and declarations, areas in Hawaii, Kentucky, Missouri, Utah, Virginia, Washington D.C., and West Virginia passed declarations after October 2020 for a total of 190 resolutions (as of March 21, 2021) (33). In addition to the geographic spread of municipalities making declarations, we observed wide variation in the declarations' content and the explanation of the role of racism in health. Many resolutions mentioned long-standing racial inequities in conditions such as cardiovascular disease, maternal, and infant mortality and more recent disparities in COVID-19, as ways that racism is reflected in health outcomes.

We also reviewed resolutions to identify specific action steps and appropriation of funding. Although a small proportion of the resolutions included action items, these are examples for how other municipalities can incorporate specific actions. In addition to the stated actions in the resolutions, Dr. Chapple-McGruder solicited responses *via* Twitter of specific action items or steps municipal governments were taking to address racism as a public health crisis or issue (Supplementary Table 1C). Although not a comprehensive review and only includes those on Twitter and willing and able to respond, some of the responses included the formation of an equity committee to execute new work, a new police oversight commission, and Washington, DC passed the REACH Act with five key components such as racial equity impact assessment, processes for accountability, and development of a commission (34, 35). Additionally, one recently published review of three declarations of racism as a public health crisis found that while the declarations are an important first step, tremendous *harm* was also done (36). This includes publicity and attention to politicians while undermining existing community or grassroots anti-racism efforts (36).

In addition to state, county, and city resolutions, there were some adopted by health departments/boards of health in states such as Arizona, Colorado, North Carolina, and Illinois (33). Also, several professional societies adopted statements and resolutions. Examples include the American Public Health Association as stated previously, the American Medical Association, and the American Psychiatric Association (37–39). The board of health and professional organizational statements and resolutions included clauses that acknowledges the history of racism in the US, denounced police brutality and recognizes the role of racism in the disproportionate impact of COVID-19 on Black, Indigenous, Latinx, and other communities of color. Some statements also urge other health and government agencies to follow suit. Similar to the other reviewed states and municipalities, most do not mention any financial considerations or explicit action steps.

After a review of the resolutions and declarations related to Racism as a Public Health Issue, there are specific recommendations that would be critical moving forward. First, extending action steps beyond training to include implementation of skills and knowledge gained is important. A majority of the resolutions call for training and the review of hiring practices in the government entities. While important, actions must go further to support communities negatively impacted by institutions with historical and contemporary practices that reinforce racism. Additionally, it is critical that those who are making decisions and in the position to implement change also have the capacity and tools to transform systems and address racism. This includes increasing the capacity of government entities and workers to be able to address racism. Another recommendation is transparency and direct partnership with communities. Communities most affected should be centered in this work, in positions to make decisions, be co-leaders and provided with the resources and funding to do this work (40–42). This includes but is not limited to community members leading in developing plans for implementing actions related to addressing racism as a public health issue as well as being allocated specific funds for any work to execute actions related to declarations of racism as a public health issue.

Additionally, these declarations and resulting actions must be intersectional and acknowledge that multiple systems of oppression converge, making the experiences of Black women for example unique. This also means being intentional about intersecting social groups and resulting hierarchies within systems related to racial and ethnic groups, immigrants, and LGBTQIA+ communities. Finally, it will also be important to hold systems accountable (including health, housing, criminal justice, and economic development) to enact change and document the impact. Currently, limited declarations have explicit language about measuring impact through data or specific accountability metrics.

## Conclusion

Given the current urgency of addressing racial inequity in the United States and specifically racial inequity in health, resolutions, and declarations naming racism as well as specific steps for undoing racism are critical. Several action steps have been noted in the current body of resolutions related to Racism as a Public Health Crisis, but more is needed. Intentional action is necessary to move the declarations from the performative space to actual anti-racism intervention. As racism was structurally embedded into systems, such as housing, interventions will need to occur at all levels, including within government and policy. Moving forward, it is clear that anti-racism action items will require the environment (e.g., “political will”) for implementation and may differ at various levels (the local, state, and national level). Further, as these are complex issues, that have existed for centuries, the financial commitment to funding anti-racism interventions will need to extend over a significant period of time. Abrupt ending to funding will only replicate the band-aid solutions that have not worked in the past. In the future, researchers could focus on understanding how racism is showing up and affecting people's lives, proximal and distal indicators that anti-racism interventions are working, and identifying and undoing other systems of oppression.

## Author Contributions

DM developed the research study, led analyses, and policy review. MM and MS were involved in the initial search and data extraction. JS, LA, and CT were involved in the secondary review, extraction, and synthesis of data. All authors were involved in writing, editing, and final approval of manuscript.

## Conflict of Interest

The authors declare that the research was conducted in the absence of any commercial or financial relationships that could be construed as a potential conflict of interest.

## Publisher's Note

All claims expressed in this article are solely those of the authors and do not necessarily represent those of their affiliated organizations, or those of the publisher, the editors and the reviewers. Any product that may be evaluated in this article, or claim that may be made by its manufacturer, is not guaranteed or endorsed by the publisher.

## References

[1] Petersen EE Davis NL Goodman D Cox S Syverson C Seed K. Racial/ethnic disparities in pregnancy-related deaths - United States, 2007-2016. MMWR Morb Mortal Wkly Rep. (2019) 68:762–5. 10.15585/mmwr.mm6835a3 31487273 PMC6730892

[2] Martin JA Hamilton BE Osterman MJK. Births in the United States, 2019. Hyattsville, MD (2020). 33054913

[3] Centers for Disease Control. U.S. Diabetes Surveillance System. (2021). Available online at: https://gis.cdc.gov/grasp/diabetes/DiabetesAtlas.html# (accessed March 19, 2021).

[4] Muntner P Carey RM Gidding S Jones DW Taler SJ Wright JT. Potential US population impact of the 2017 ACC/AHA High Blood Pressure Guideline. Circulation. (2018) 137:109–18. 10.1161/CIRCULATIONAHA.117.032582 29133599 PMC5873602

[5] Yoon SSS Carroll MD Fryar CD. Hypertension prevalence and control among adults: United States, 2011-2014. NCHS Data Brief. (2015) 220:1–8. Available online at: https://www.cdc.gov/nchs/data/databriefs/db220.pdf 26633197

[6] Van Dyke ME Mendoza MCB Li W Parker EM Belay B Davis EM. Racial and ethnic disparities in COVID-19 incidence by age, sex, and period among persons aged <25 years - 16 U.S. Jurisdictions, January 1-December 31, 2020. MMWR Morb Mortal Wkly Rep. (2021) 70:382–8. 10.15585/mmwr.mm7011e1 33735165 PMC7976617

[7] Gilmore RW. Geographies of Global Change: Remapping the World, 2nd Edn. Malden, MA: Blackwell Publishing (2002). 261 p.

[8] Paradies YC. Defining, conceptualizing and characterizing racism in health research. Crit Public Health. (2006) 16:143–57. 10.1080/09581590600828881

[9] Jones CP. Toward the science and practice of anti-racism: launching a National Campaign against racism. Ethn Dis. (2018) 28(Suppl. 1):231–4. 10.18865/ed.28.S1.231 30116091 PMC6092166

[10] Jones CP. Levels of racism: a theoretic framework and a gardener's tale. Am J Public Health. (2000) 90:1212–5. 10.2105/AJPH.90.8.1212 10936998 PMC1446334

[11] Geronimus AT Hicken M Keene D Bound J. “Weathering” and age patterns of allostatic load scores among blacks and whites in the United States. Am J Public Health. (2006) 96:826–33. 10.2105/AJPH.2004.060749 16380565 PMC1470581

[12] Clark R Anderson BA Clark VR Williams DR. Race, Ethnicity, and Health: A Public Health Reader, 2nd Edn. San Francisco, CA: Jossey-Bass (2012). p. 79–104.

[13] Bailey ZD Krieger N Agénor M Graves J Linos N Bassett MT. Structural racism and health inequities in the USA: evidence and interventions. Lancet. (2017) 389:1453–63. 10.1016/S0140-6736(17)30569-X 28402827

[14] Williams DR. Racial residential segregation: a fundamental cause of racial disparities in health. Public Health Rep. (2001) 116:404–16. 10.1016/S0033-3549(04)50068-7 12042604 PMC1497358

[15] Cell JW. The Highest Stage of White Supremacy: The Origins of Segregation in South Africa and the American South. New York, NY: Cambridge University Press (1982). p. 15–320.

[16] Massey DS. American apartheid: segregation and the making of the underclass. Am J Sociol. (1990) 96:329–57. 10.1086/229532

[17] National Research Council. A Common Destiny: Blacks and American Society. Washington, DC: National Academies Press (1989).

[18] Mendez DD Hogan VK Culhane J. Institutional racism and pregnancy health: using Home Mortgage Disclosure Act data to develop an index for mortgage discrimination at the community level. Public Health Rep. (2011) 126:102–14. 10.1177/00333549111260S315 21836743 PMC3150135

[19] Gee GC. A multilevel analysis of the relationship between institutional and individual racial discrimination and health status. Am J Public Health. (2002) 92:615–23. 10.2105/AJPH.92.4.615 11919062 PMC1447127

[20] Beyer KMM Zhou Y Matthews K Bemanian A Laud PW Nattinger AB. New spatially continuous indices of redlining and racial bias in mortgage lending: links to survival after breast cancer diagnosis and implications for health disparities research. Health Place. (2016) 40:34–43. 10.1016/j.healthplace.2016.04.014 27173381

[21] Lukachko A Hatzenbuehler ML Keyes KM. Structural racism and myocardial infarction in the United States. Soc Sci Med. (2014) 103:42–50. 10.1016/j.socscimed.2013.07.021 24507909 PMC4133127

[22] Wallace ME Mendola P Liu D Grantz KL. Joint effects of structural racism and income inequality on small-for-gestational-age birth. Am J Public Health. (2015) 105:1681–8. 10.2105/AJPH.2015.302613 26066964 PMC4504298

[23] Williams DR Lawrence JA Davis BA. Racism and health: evidence and needed research. Annu Rev Public Health. (2019) 40:105–25. 10.1146/annurev-publhealth-040218-043750 30601726 PMC6532402

[24] Wallace M Crear-Perry J Richardson L Tarver M Theall K. Separate and unequal: structural racism and infant mortality in the US. Health Place. (2017) 45:140–4. 10.1016/j.healthplace.2017.03.012 28363132

[25] Chambers BD Baer RJ McLemore MR Jelliffe-Pawlowski LL. Using index of concentration at the extremes as indicators of structural racism to evaluate the association with preterm birth and infant mortality-California, 2011-2012. J Urban Heal. (2019) 96:159–70. 10.1007/s11524-018-0272-4 PMC6458187 29869317

[26] Anti-Racism in Public Health Act of 2020. H.R., 8178, 116th Congress (2020).

[27] Black Maternal Momnibus Act 2020. H.R., 6142, 116th Congress (2020).

[28] McFarlane AC Norris FH. Definitions and concepts in disaster research. In: Norris F, Galea S, Friedman M, Watson P, editors. Methods for Disaster Mental Health Research. New York, NY; London: Guilford Press (2006). p. 13–9.

[29] Boston University School of Public Health. Crying Crisis. (2017). Available online at: https://www.bu.edu/sph/news/articles/2017/crying-crisis/ (accessed March 26, 2021).

[30] Health Equity Solutions. Declaring Racism a Public Health Crisis in Connecticut. (2021). Available online at: https://www.hesct.org/blog/declaring-racism-public-health-crisis-connecticut/ (accessed March 16, 2021).

[31] A Resolution Requesting Approval to Recognize April 1-7 as National Public Health Week and Supporting Milwaukee County's Commitment to Achieve Racial Equity and Transform Systems and Institutions Impacting the Health of Our Community 19-397 Resolution Milwaukee County (2019).

[32] Ford CL Airhihenbuwa CO. 2010 The public health critical race methodology: praxis for antiracism research. Soc Sci Med. (1982) 71:1390–8. 10.1016/j.socscimed.2010.07.030 20822840

[33] American Public Health Association. Racism as a Public Health Crisis. (2021). Available online at: https://www.apha.org/topics-and-issues/health-equity/racism-and-health/racism-declarations (accessed March 25, 2021).

[34] Twitter. Theresa Chapple. (2021). Available online at: https://twitter.com/Theresa_Chapple/status/1352409426020491268?s=19 (accessed February 23, 2021).

[35] Kenyan McDuffie Ward 5. The Reach Act- The Racial Equity Achieves Results Act of 2020. (2021). Available online at: https://kenyanmcduffieward5.com/equity/ (accessed March 25, 2021).

[36] Paine L De La Rocha P Eyssallenne AP Andrews CA Loo L Jones CP. Declaring racism a public health crisis in the United States: cure, poison, or both? Front Public Health. (2021) 9:606. 10.3389/fpubh.2021.676784 PMC8265203 34249843

[37] American Public Health Association. Structural Racism is a Public Health Crisis: Impact on the Black Community. (2021). Available online at: https://www.apha.org/policies-and-advocacy/public-health-policy-statements/policy-database/2021/01/13/structural-racism-is-a-public-health-crisis (accessed March 25, 2021).

[38] American Medical Association. AMA: Racism is a Threat to Public Health. (2020). Available online at: https://www.ama-assn.org/delivering-care/health-equity/ama-racism-threat-public-health (accessed March 25, 2021).

[39] American Psychiatric Association. APA's Apology to Black, Indigenous and People of Color for Its Support of Structural Racism in Psychiatry. (2022). Available online at: https://www.psychiatry.org/newsroom/apa-apology-for-its-support-of-structural-racism-in-psychiatry (accessed March 25, 2021).

[40] Ford CL Airhihenbuwa CO. Critical race theory, race equity, and public health: toward antiracism praxis. Am J Public Health. (2010) 100:S30–S5. 10.2105/AJPH.2009.171058 20147679 PMC2837428

[41] Arnstein S. A ladder of citizen participation. Am Inst Plann J. (1969) 35:216–24. 10.1080/01944366908977225

[42] Scott KA Bray S McLemore MR. First, do no harm: why philanthropy needs to re-examine its role in reproductive equity and racial justice. Health Equity. (2020) 4:17–22. 10.1089/heq.2019.0094 32219193 PMC7097698

