# Supplementary material for: Racism as Public Health Crisis: Assessment and Review of Municipal Declarations and Resolutions Across the United States
Source: Front Public Health. 2021 Aug 11;9:686807. doi: 10.3389/fpubh.2021.686807 (PMC8385329; doi:10.3389/fpubh.2021.686807)
Supplement: Supplementary file 1 [file Table_1.DOCX]

**Supplementary Table 1A: Extensive Table of declarations, resolutions and relevant websites for content**

|  |  |  |  |  |  | **Legislation includes historical context specific to** | | |  |  |  | **Maternal Child Health (MCH)** | | **Living Wage / Universal Basic Income (UBI)** | |  |  |  |  |  |  |  |  |  |  |
| --- | --- | --- | --- | --- | --- | --- | --- | --- | --- | --- | --- | --- | --- | --- | --- | --- | --- | --- | --- | --- | --- | --- | --- | --- | --- |
| **State** | **Location**  **(e.g. City or County)** | **Date Submitted** | **Date Approved** | **Background of Legislation (process)** | **Name and title of person**  **who introduced the legislation** | **City or County (y/n)** | **Unique examples of historical context City/County level** | **State (y/n)** | **Unique examples of historical context State level** | **National (y/n)** | **Unique examples of historical context**  **National level** | **Mentions MCH (y/n)** | **Describe what legislation says about MCH or proposes to do** | **Mentions living wage or UBI (y/n)** | **Describe what aspects of living wage, UBI, or similar policy is discussed** | **Major component(s) of legislation** | **Themes** | **Action** | **Is funding**  **discussed (y/ n)** | **Describe what aspects of funding are discussed.** | **Any organizations or**  **entities mentioned? Include names here.** | **Comment if any unique or**  **notable aspects of the legislation (can be pos or neg)** | **Additional links or websites related to the legislation** | **Another link or website related to the legislation** | **Official Resolution** |
| AR | Fayetteville |  | 8/18/2020 | Introduced by African American Advisory  Council and approved by Mayor Lioneld Jordan on 8/18/20 | D’Andre Jones (Mayors  African American Advisory Council member) | N |  | N |  | Y |  | Y | high infant and maternal mortality rates is an example of a worse health outcome for African  Americans | N |  | Defining racism along with its national  history and impacts on communities of color; action steps | Systemic Racism Interpersonal Racism Health outcomes SDOH  COVID-19  Center Community experiences and voices  Criminal Justice system and prison industrial complex Racial Justice  Police Reform | Actions related to annual report of racial disaggregated data, community development/investment, police reform, and racially equitable policies; also action to develop a  racial equity action plan | Y | Commitment to increase  funding for public recreation resources |  |  |  |  | <http://documents.fayetteville-ar.gov/WebLink> |
| CA | Coachella | 7/22/2020 | 7/22/2020 | Unanimously passed 5-0 | City Council | N |  | N |  | Y |  | N |  | N |  | Acknowledging those who were lost due to racist police violence; Acknowledging racism's historical and present implications and impacts for  communities of color; Action steps | systemic racism  criminal justice system and prison industrial complex police violence  COVID-19 SDOH  racial justice | stand in solidarity, call for systemic change to eliminate barriers; advance justice, equity, diversity, and inclusion; end racial and social disparities; expand community voice and power; partner with others to close gap of racial injustice; establish ad hoc Special Equity and Social Justice  Committee; regular reporting | N |  |  |  |  |  | https://mccmeetingspublic.blob.core.usgovcl |
| CA | Fontana | 7/14/2020 | 7/14/2020 | Motion was made by Mayor Pro Tem  Armendarez, passed unanimously by a vote of 5-0-0 to adopt | Mayor Pro Tem Jesse Armendarez | N |  | N |  | N |  | N |  | N |  | Recognition of disparities as a result of racism; health and societal consequences of racism; City racial  demographics and data; actions | systemic racism interpersonal racism health outcomes  criminal justice and prison industrial complex  COVID-19 | initiate and support efforts that work towards promoting fair and just society; eliminate barriers; | N |  | World Health Organization |  | https://[www.fontana.org/ArchiveC](http://www.fontana.org/ArchiveC) |  | https://[www.fontana.org/DocumentCenter/V](http://www.fontana.org/DocumentCenter/V) |
| CA | Goleta, CA |  | 6/8/2020 | Not found | Councilmember Kyle  Richards (per newspaper article by J. Yamamura) | Y | Includes name of first black  enslaved resident in that county. | N |  | Y |  |  |  |  |  |  | Black Lives Matter | Declaration of racism as a public health emergency |  |  |  | No actions listed at all | https://[www.independent.com/20](http://www.independent.com/20) |  | https://content.govdelivery.com/attachments |
| CA | Indio | 8/19/2020 | 9/1/2020 | issues discussed in 8/19/20 meeting and resolution was recommended; resolution  presented and approved 9/1/20 | Mayor Pro Tem Elaine Holmes and Councilmember Oscar  Ortiz | N |  | N |  | N |  | N |  | N |  | Acknowledge the death of those who were murdered by the hands of police  violence; | systemic racism interpersonal racism criminal justice system and prison industrial complex SDOH  health equity | governmental organization; increase diversity  across City workforce and leadership; review all policies, procedures, and practices to ensure racial equity; identify and implement solutions to eliminate systemic inequality; enhance public education; implement community-based alternatives to address harms and prevent trauma; advocate for relevant local, state, and federal policies that improve health in communities of color partner to assess and apply and equity lens to  internal policies | N |  | U.S. National Institutes of Health |  | https://[www.indio.org/civicax/fileb](http://www.indio.org/civicax/fileb) | https://[www.desertsun.com/story/](http://www.desertsun.com/story/) | https://[www.indio.org/civicax/filebank/blobd](http://www.indio.org/civicax/filebank/blobd) |
| CA | Los Angeles City Council, CA |  | 6/9/2020 |  | Supervisor Mark Ridley- Thomas | Y |  | N |  | N |  |  |  |  |  |  | health outcomes SDOH | Assess policy and procedures including budget process, Support community efforts to amplify issues around racism, Encourage racial equity training among community partners, grantees,  vendors, identify clear goals to advance racial equity |  |  |  | Board directed the county's CEO to come up with a strategic plan to prioritize health, housing, etc. for Black residents. The same day they also passed a measure to allow voters to weigh in on  county's community investment | https://losangeles.cbslocal.com/20 |  | https://clkrep.lacity.org/onlinedocs/2020/20- |
| CA | Long Beach | 6/5/2020 | 6/9/2020 | not found | Councilmember Rex Richardson | N |  | N |  | Y |  | Y | 1/4 of very low birth weight babies are born to African  American mothers | N |  | History/background of racism in the US and impacts today  Examples of inequalities/the realities of being black in the city  Action steps | experiences and voices police violence  COVID-19  racial justice redlining  health outcomes  criminal justice system and prison industrial complex racial disparities  systemic racism | listening to experiences of impacted community members; convening discussions with communities of color and other community stake holders; call for budget investment; law enforcement reform; apply the City's Equity Tool Kit to internal decisions to city council; ensure the diverse community  reflect city government; review existing reform | Y | Calls for the city to budget investment in housing, jobs, education, youth development, health care, community centers,  and open spaces | Reconciliation Commissions (Canada and Australia) | this resolution is described with a "Framework for Reconciliation" (see additional links); Sites Truth and Reconciliation Commissions in Canada and Australia (and their roles in post-apartheid South  Africa) | <http://www.longbeach.gov/health/> | https://[www.presstelegram.com/2](http://www.presstelegram.com/2) | https://longbeach.legistar.com/View.ashx?M= |
| CA | Moreno Valley | 7/7/2020 | 7/7/2020 | unanimously voted to support | Council Member Thornton and Mayor Gutierrez | N |  | N |  | N |  | N |  | N |  | n/a | n/a | n/a | N |  |  |  | https://morenovalleyca.iqm2.com/ | https:// morenovalleyca.iqm2.com/ Citizens/FileOpen.aspx?  Type=15&ID=1966&Inline=True | <http://morenovalleyca.iqm2.com/Citizens/File> |
| CA | Oxnard | 6/30/2020 | 6/30/2020 | Request of resolution by Councilmember Lopez and Mayor Pro Tem Ramirez at previous meeting; presented at 6/30 meeting and voted  7-0 | Councilmember Lopez | N |  | N |  | Y |  | Y | People of color experience increased rates of infant mortality | N |  | History/background of racism in the US and impacts today  Action steps | police violence COVID-19  systemic racism redlining  criminal justice system and  prison industrial complex | condemning police brutality and hold police departments accountable for their actions; Improvement of Police department policies and practices; calls for anti-racist law enforcement  policies that address police brutality | N |  | CDC |  | https://[www.vcstar.com/story/new](http://www.vcstar.com/story/new) | https://oxnardca.civicclerk.com/W | ehttps://oxnardca.civicclerk.com/Web/GenFile |
| CA | Palm Springs | 8/6/2020 | 8/6/2020 | listening session on 7/18/20; resolution passed unanimously 5-0 on 8/6/20 | Councilmember Grace Elena Garner | N |  | N |  | N |  | N |  | N |  | Defining racism along with its impacts on communities of color; action steps | systemic racism  criminal justice system and prison industrial complex COVID-19  SDOH  healthcare access  centering community experiences and voices | Commitment to meaningfully advance justice, equity, diversity, and inclusion;  intend to end racial and social disparities; expand community voice and power to close gap  of racial injustice and better serve communities of color | N |  |  |  | https://[www.palmspringsca.gov/](http://www.palmspringsca.gov/)  home/showpublisheddocument? id=76870 | https://[www.palmspringsca.gov/Ho](http://www.palmspringsca.gov/Ho) | https://[www.palmspringsca.gov/home/showp](http://www.palmspringsca.gov/home/showp) |
| CA | Redlands | 7/21/2020 | 7/21/2020 | 4-0 in favor of resolution | Mayor Pro Tem Denise Davis and Councilman  Eddie Tejeda | N |  | N |  | Y |  | N |  | N |  | Defining racism and describing its impact historically and today in Black and Native American communities; The cities relevant past work; Action  Steps | health outcomes systemic racism  criminal justice system and prison industrial complex centering community  experiences and voices | endorse continued policies and practices for employee conduct and equitable treatment; active dismantling of remnants of racism; annual training, revising department policies; give people of color opportunities in hiring processes; diversify race, gender, age in city commission; report progress; support community effort; adding health, equity, and justice as objectives; support policies and  other organizations | N |  | United States Office of Disease Prevention;  U.S. Census Bureau |  | https://destinyhosted.com/agenda |  | https://destinyhosted.com/redladocs/2020/C |
| CA | Rialto | 7/28/2020 | 7/28/2020 | Motion by Council Member Carrizales and second by Council Member  Trujillo and carried by a 5-0 vote to Adopt | City Manager Rod Foster | N |  | N |  | N |  | Y | maternal death and premature birth stem from racism; infant mortality rate with San Bernardino County's Black  population is more than double the rate for the County as a whole | N |  | Defining racism along with its  implications and impacts for communities of color; action steps | health outcomes  criminal justice system and prison industrial complex healthcare access  centering community experiences and voices | Volunteer to serve on the County's Equity Element Group while collaborating with them and  reviewing/considering their policies; enhance diversity in City's workforce; Advocate through the League of California Cities, National League of Cities, US Conference of Mayors, and GARE; Support Community Efforts; Review County's existing policies through lens of racial equity to  promote policies that prioritize health; Encourage Cities and Towns in County to adopt resolutions | N |  | United States Office of Disease Prevention  U.S. Census Bureau  GARE (Government Alliance on Race and Equity) |  | https://rialto.legistar.com/Legislati |  | https://rialto.legistar.com/LegislationDetail.as |
| CA | Riverside City | 6/26/2020  (on agenda) | 6/30/2020 | Approved on 6/30/20 meeting following discussion by councilmembers | Councilwoman Plascencia | N |  | N |  | N |  | N |  | N |  | Commitments to actions by city council | n/a | Institute racial equity training  enhance diversity and anti-racism principles; promote equity through all policies; advocate at local, state, federal level for relevant policies; support community efforts of issues of racism;  partner with other organizations confronting racism | N |  |  |  | https://riversideca.legistar.com/Vie | https://riversideca.legistar.com/Vie | https://riversideca.legistar.com/View.ashx?M |
| CA | Riverside County | 7/31/2020 | 8/4/2020 | Submitted to the board of supervisors; approved by board at 8/4/20 meeting | Supervisor Chuck  Washington and Supervisor V Manuel Perez | N |  | N |  | N |  | N |  | N |  | The role of racism on county residents;  County's commitments of actions to address racism | systemic racism task force  criminal justice system and prison industrial complex SDOH  health equity | Create governmental awareness and increase equity, diversity and public health; work with the department of public health, county departments  and community partners to apply equity lens on policies, practices, and preventative measures | N |  |  |  |  |  | https://kesq.b-cdn.net/2020/08/Snapshot-914 |
| CA | San Bernardino County |  | 6/23/2020 |  | Supervisor Janice Rutherford | Y |  | N |  | Y |  | Y | 1. Maternal death is listed in a list of health conditions "stemming from racism". 2. The infant mortality rate among the county's  Black pop'n is more than double the rate for the County. |  |  |  | redlining Covid-19 police violence  health outcomes | Collaborate with law and justice agencies to ensure public's confidence. Promote equity through policies Identify activities to enhance diversity  withing workforce Advocate through Ass'n of  Counties for policies to improve health outcomes...  Strengthen alliances with CBO which are confronting racism, |  |  | proposed membership in Gov't Alliance on Race and Equity (GARE) - a nat'l network of local gov't.  https://www.racialequityalli ance.org/ | Provides bulleted list of things that the county will do to "actively participate in the dismantling of racism". 1st is add "Equity" as the 11th element within the Countywide vision. Not reflected  on website yet as of 12.16.20 | <http://cms.sbcounty.gov/cao-vision> |  | https://sanbernardino.legistar.com/View.ashx |
| CA | San Luis Obispo | 6/16/2020 | 6/16/2020 | Unanimously adopted 6/16/20 by city council | City Manager Derek Johnson | N |  | N |  | Y |  | N |  | N |  | Historical context of racism in is the US;  Current impact of racism for people of color in; actions | COVID-19  criminal justice system and prison industrial complex police violence  hate crimes  Black Lives Matter | make city a welcoming safe and inclusive community condemning racism, violence, and hate; stand in solidarity with Black Lives Matter  movement | Y | Funding not mentioned in resolution, but a month before resolution the city council  allocated $160,000 for diversity and inclusion |  |  | https://opengov.slocity.org/WebLi | https://[www.sanluisobispo.com/ne](http://www.sanluisobispo.com/ne) | https://opengov.slocity.org/WebLink/DocVie |
| CA | Santa Barbara | 6/23/2020 | 6/23/2020 | Unanimously adopted 6/23/20 by city council | Organization- Healing Justice: Black Lives Matter Santa Barbara | Y | Includes name of first black enslaved resident in that county | N |  | Y |  | N |  | N |  | Acknowledges the recent killings of black people at the hands of police  violence; current impact of racism on communities of color; actions | COVD-19  Black Lives Matter police Violence  criminal justice system and prison industrial complex | Urge Police department to commit to fair and impartial policing policies and practices; establishing an independent civilian police oversight system; condemn any and all police  brutality | N |  |  | specifically mentions LGBTQ+, immigrants, and those with disabilities as marginalized people | https://[www.independent.com/20](http://www.independent.com/20) |  | https://records.santabarbaraca.gov/OnBaseAg |
| CA | Santa Clara County | 6/23/2020 | 6/23/2020 | unanimously adopted resolution 6/23/2020 | Supervisor Dave Cortese | N |  | N |  | Y |  | Y | infant mortality is a negative repercussion of historical racism | N |  | Historical context of racism in is the US; Current impact of racism for people of color in; county's commitments and  actions | systemic racism health outcomes police violence racial disparities SDOH  redlining COVID-19 | 11 action steps related to educational efforts, community engagement, looking at policies through racial equitable lens, training and hiring practices, and encouraging other organizations, cities, states, and federal entities to take similar  actions and declare racism as a public health crisis | Y | Number 11 of the explicit action steps relates to "securing adequate resources to  successfully accomplish" the other action steps |  | This resolution was made in conjunction with a resolution affirming the Black Lives Matter  and condemning racial violence | <http://sccgov.iqm2.com/Citizens/Fi> | <http://sccgov.iqm2.com/Citizens/ca> | <http://sccgov.iqm2.com/citizens/FileOpen.asp> |
| CA | Santa Cruz County | 8/12/2020 | 8/18/2020 | On agenda 8/12/20; Unanimously approved at 8/18/20 county supervisors meeting | John Leopold (First District Supervisor) Ryan Coonerty  (Third District Supervisor) | N |  | N |  | Y |  | Y | Infant Mortality is a negative health outcome of historical  racism | N |  | Define racism and consequences for communities of color; Action steps | systemic racism racial disparities health outcomes police violence redlining  COVID-19  centering community experiences and voices | Incorporating educational efforts; promoting community engagement; review ordinances and human resources practices through a racial equity lens; promoting racial equitable economic; promoting policies that prioritize health of people of color; training of elected officials; partnering and building alliances with organizations confronting racism and encourage and community partners to confront racism; support local, state, and federal initiatives to advance social justice and combat  racism; | N |  | United States Office of Disease Prevention |  | https:// [www.santacruzsentinel.com/](http://www.santacruzsentinel.com/) 2020/08/18/second-pandemic- santa-cruz-county-declares-  racism-a-public-health-crisis/ |  | https://santacruzcountyca.iqm2.com/citizens/ |
| CA | Ventura City | 7/10/2020 | 7/13/2020 | Councilmember Brown shared her experience with bias in policing in 6/29/2020 meeting and made motion for a resolution; resolution  passed by unanimous vote on 7/13/2020 | City Councilmember Lorrie  Brown and Deputy Mayor Sofia Rubalcava | N |  | N |  | Y |  | N |  | N |  | Acknowledge victims of police brutality  and racism; acknowledge the history and impacts of racism; action steps | Black Lives Matter  criminal justice system and prison industrial complex healthcare access  police violence | declare black lives do matter; condemn police brutality; adopt Mayor's statement regarding "#8CANTWAIT" (police reform); combat explicit and implicit racism in hiring practices and creating diversity in organization; use city funds; police and criminal justice reform (excessive force, hiring  practices, and school-to-prison pipeline specifically); invest in economic development | Y | Pledge to use city funds to  partner with county, public health and non profits |  | Acknowledges the queer/transgender people of color as well as gender inequality ,  homophobia, the disabled, and immigrants |  |  | https://[www.cityofventura.ca.gov/Document](http://www.cityofventura.ca.gov/Document) |

| CA | Yolo County | 7/21/2020 | 7/21/2020 | unanimously passes and adopted 7/21/20 | Gary Sandy, Chair of Yolo County Board of  Supervisors | N |  | N |  | N |  | N |  | N |  | acknowledging the issues of racism and describing its impacts on Black and Latinx people; commitment to  action | healthcare access health equity  criminal justice system and  prison industrial complex | vague mention of "Course of action" | N |  | United States Office of Disease Prevention |  |  |  | https://yoloagenda.yolocounty.org/docs/2020 |
| --- | --- | --- | --- | --- | --- | --- | --- | --- | --- | --- | --- | --- | --- | --- | --- | --- | --- | --- | --- | --- | --- | --- | --- | --- | --- |
| CO | Denver, CO |  | 6/8/2020 | COMMENTS ABOUT THE PROCESS AT THE DEPT  OF PH... NOT CITY Employees at CO Dept PH  pushed the executive director to make a statement. Initial response was a letter stating racism as a "critical public health issue". Letter found here: https://covid19.colorado.gov/blog- post/open-letter-racism-is-a-persistent-and- critical-public-health-issue  Employees continued to push until resolution achieved by Denver Dept PH on 8/13/20.  https://[www.denverpost.com/2020/07/31/](http://www.denverpost.com/2020/07/31/) colorado-racism-public-health-crisis/ | A motion offered by Councilmember Gilmore, duly seconded by Councilmember Flynn, that Council Proclamation  be adopted. 12 yes, 1 person absent | Y | Denver’s legacy of redlining is well-documented, ... neighborhoods that were redlined have higher poverty rates, lower social mobility, more indicators of social decline, and when mapped have long been  referred to as Denver’s inverted-L, " | N |  | Y | "WHEREAS, racism is rooted in the foundation of America, beginning  with chattel slavery in 1619;" | N |  | N |  | Historical context is strong, also link with economic factors, and health impacts. Actions are broad, note action to involve all residents in racial justice work and a statement about providing tools to members of local gov't to enable them to engage actively and authentically with communities of color... both  statements speak to community involvement | SDOH  Redlining COVID-19  police violence health outcomes racial justice | City responsibility to seek "solutions to reshape the discourse and actively engage all residents in racial justice work"  Advocate for racial justice as a core element of Denver’s policies, programs and procedures.  Support the expansion of documented equity decision-making frameworks  Agency organizational work plans to address and correct embedded policies that discriminate and perpetuate racism  Educational efforts to address and dismantle racism | N |  | 10/2020 Not mentioned in the resolution, but the health system also did a proclamation. Article states that Denver Health joined 38 other health systems...  https:// [www.denverhealth.org/](http://www.denverhealth.org/)  news/2020/10/racism-is-a- public-health-crisis | Defines public health crisis. "although there is no epidemiologic definition of “public health crisis,” the health impacts of racism clearly exemplify the definition proposed by experts from the BUSPH which is that “The problem must affect large numbers of people, it must threaten health over the long-  term, and it must require the adoption of large-scale solutions” | https://denver.legistar.com/View.a | DENVER board of Public Health. https://[www.denvergov.org/cont](http://www.denvergov.org/cont) ent/dam/denvergov/Portals/771/ documents/BEH/ Racism_PublicHealthDeclaration_ BPHErecommendation.pdf  Conservative newspaper post Nov 2020 Racism is not a PH crisis.  Shooting heroine and people bathing in the park are real crises. https://thecoloradoherald.com/20  20/denver-declares-racism-a- public-health-crisis/ | https://denver.legistar.com/View.ashx? M=F&ID=8585802&GUID=25C9ACF9-7B6E- 479B-89BB-BED8E9F36CC6 |
| CT | Bloomfield, CT |  | 6/22/2020 |  |  | N |  | N |  | N |  | N |  | N |  | mentions dimensions of racism, race is  social construct, and no biological basis, | COVID-19  health outcomes racial disparities | Improve quality of data the city collects, make alliances with organizations also ding this work, identify goals and objectives to assess progress to  advance racial equity | N |  |  |  |  |  | https://[www.bloomfieldct.gov/sites/g/files/vy](http://www.bloomfieldct.gov/sites/g/files/vy) |
| CT | Bridgeport | 7/21/2020 | 7/21/2020 | Motion Massed Unanimously | Council Member Cruz | N |  | N |  | N |  | Y | maternal death and premature birth stem from racism | N |  | Defining racism along with it's  implications and impacts for communities of color; action steps | systemic racism interpersonal racism criminal justice system and prison industrial complex SDOH  health outcomes  healthcare access COVID-19 | enhance diversity to progress equity and justice oriented organization; promote equity through all policies to dismantle systemic racism; improving quality of data collected; advocate locally for relevant policies; solidify alliances and partnerships with other organisms fighting racism; support community efforts to amplify issues of racism;  identify clear goals and periodic reports | N |  | United States Office of Disease Prevention |  |  |  | https://[www.bridgeportct.gov/filestorage/](http://www.bridgeportct.gov/filestorage/) 87203/97755/362737/362741/2020-07-  21.pdf |
| CT | Colchester | 7/16/2020 | 7/16/2020 | Motion Carried 4-1 | First Selectman (FS) Mary Bylone and Selectman  Denise Turner | N |  | N |  | N |  | N |  | N |  | n/a | n/a | n/a | N |  |  |  |  |  | Only Able to Access Meeting Minutes:  https://[www.colchesterct.gov/sites/g/files/](http://www.colchesterct.gov/sites/g/files/) vyhlif4286/f/minutes/  board_of_selectmen_meeting_0.pdf |
| CT | Easton | 8/20/2020 | 8/20/2020 | Motion passed unanimously | Selectmen David Bindelglass | N |  | N |  | N |  | N |  | N |  | Defining racism along with present consequences for people of color;  Action Steps | systemic racism interpersonal racism health outcomes COVID-19  task force  centering community experiences and voices | Enhance diversity to ensure antiracism principles through activities; Promote racial and health equity in policies; strive to improve quality of data; advocate for relevant policies that improve health; support local, state, regional and federal initiatives; solidify alliances and partnerships and partnerships with other organizations; support community efforts  to amplify issues of racism | N |  |  |  |  |  | https://[www.eastonct.gov/sites/g/files/](http://www.eastonct.gov/sites/g/files/) vyhlif3071/f/minutes/ board_of_selectmen_regular_meeting_minu  tes_08-20-2020.pdf |
| CT | Glastonbury | 7/28/2020 | 7/28/2020 | Motion passed unanimously 9-0 | Council Members Deborah Carroll and Lillian Tanski | N |  | N |  | Y |  | N |  | N |  | Defining racism along with its impacts on communities of color; action steps | criminal justice system and prison industrial complex COVID-19  racial justice centering community  experiences and voices  police reform | Appoint a Commission on Racial Justice; Hold hearings and collect data on minority residents' experiences of policing, education, and recreation; affirm rights to belong for residents and visitors especially in public and recreational spaces; Police reports on measures to ensure fair treatment including hiring and training; identify opportunities  to enhance diversity | N |  |  |  | https://[www.glastonburyct.gov/Ho](http://www.glastonburyct.gov/Ho) |  | https://[www.glastonbury-ct.gov/Home/Show](http://www.glastonbury-ct.gov/Home/Show) |
| CT | Hamden | 7/17/2020 | 7/20/2020 | First presented by Mayor to Hamden Legislative Council on 7/17/20; Voted unanimously 7/20/20 by legislative council | Mayor Curt Balzano Leng | N |  | N |  | N |  | Y | racism has caused a disproportionate burden of infant mortality | N |  | Defining racism and its impact on Black, Native American, Asian, and Latino residents; action steps | systemic racism interpersonal racism SDOH  criminal justice system and prison industrial complex COVID-19  health outcomes | enhance diversity to progress equity and justice oriented organization; promote equity through all policies to dismantle systemic racism; improving quality of data collected; advocate locally for relevant policies; solidify alliances and partnerships with other organisms fighting racism; support community efforts to amplify issues of racism;  identify clear goals and periodic reports | N |  |  |  | https://[www.wtnh.com/news/conn](http://www.wtnh.com/news/conn) |  | https://[www.hamden.com/DocumentCenter/](http://www.hamden.com/DocumentCenter/) |
| CT | Hartford | 6/22/2020 | 6/22/2020 | Unanimously Endorsed after unanimous vote  on resolution to defund police and recognize Juneteenth a holiday | City Council Endorsement | N |  | N |  | N |  | N |  | N |  | n/a | police violence | n/a | N |  |  |  | https://[www.courant.com/commu](http://www.courant.com/commu) | https://ctmirror.org/2020/06/24/ connecticut-towns-are-declaring- racism-a-public-health-crisis-  advocates-want-the-state-to- follow/ | Only Able to Access News Article:  https://[www.courant.com/community/](http://www.courant.com/community/) hartford/hc-news-har-police-budget-  20200623-u5vyxekadfak3is6uzdc2w7tvy- story.html |
| CT | Manchester |  | 7/7/2020 | 9 voted in favor (unanimous) | Introduced by Sarah Jones  (Deputy mayor) and Tim Bergin (Board member) | N |  | N |  | Y | broad statements of the disparities, nothing unique | N |  | N |  | Describes the problem, with little info on action | health outcomes racial disparities COVID-19  SDOH | Board of Directors of the Town "establish itself as an equity and justice-oriented organization"  Educational efforts to understand how racism "  affects the delivery of human and social services, economic development and public safety" | N |  |  | Intro notes race as social construct, and includes the levels of racism (interpersonal, systemic, etc.)  Most of the CT resolutions are almost verbatim the same  Odd that no details of discussion in the minutes. Several other topics have a description of the discussion, but nothing provided  for the resolution of racism as a PH crisis. | bod.townofmanchester.org/NewBO |  | <http://bod.townofmanchester.org/NewBOD/a> |
| CT | New Britain |  | 6/24/2020 |  | Not sure...  Ald. Osborn moved to accept and adopt, seconded by Ald.  Santiago | N |  | N |  | Y | Same language as other cities in CT | Y | "Disproportionate burden of illness and mortality including COVID-19 infection and death, heart disease, diabetes, and  infant mortality" | N |  | Describes manifestations of racism- poverty, health outcomes, access to care, etc.  Vague actions | health outcomes racial disparities COVID-19  SDOH | Similar language to other cities in CT.  City of New Britain will work to progress as an equity and justice-oriented organization, by continuing to identify specific activities to enhance diversity and to ensure antiracism principles across our leadership, staffing and contracting ....  language present in other CT  Enhance educational efforts aimed at understanding and dismantling racism  Improve quality of data collected same as other cities in this state. | N |  | N | Newspaper article publishing about the resolution passing mentioned a local org: Health Equity Solutions  https://[www.hesct.org/](http://www.hesct.org/) | https://newbritain.granicus.com/D |  | https://legistarweb-production.s3.amazonaws |

| CT | New Haven |  | 7/7/2020 | passed unanimously | Darryl Brackeen Jr. (alderman) and Tyisha Walker-Myers (board of  Alders president) | N |  | N |  | Y | just broad statements of  the disparities, nothing unique | N |  | N |  | Quote is from a news article... "New Haven’s declaration outlines the city’s need to identify activities to enhance diversity and anti-racism principles in its own leadership, staffing and contracting; advance educational efforts on understanding and dismantling racism; boost data collection; support policy that improves health in communities of  color; and take other steps to advance racial equity" | COVID-19  health equity  racial disparities SDOH | Working group created the same evening, and another resolution to the New Haven delegation to the General Assembly to end gerrymandering related to incarcerated residents. Meeting minutes not available, but these items were noted as done on the agenda.  Also, newspaper on 7/2 said that aldermen were appointing the 5 people to the Civilian Review Board at the 7/6 meeting. Again, no meeting minutes avail. Seems linked to the resolution of Racism as PH Crisis, but not mentioned in the actual resolution.  https://[www.newhavenindependent.org/index.ph](http://www.newhavenindependent.org/index.ph) p/archives/entry/crb_nominees/ | N |  |  | Wording is almost verbatim of the Bloomington, CT proclamation. | https://[www.wnpr.org/post/conn-c](http://www.wnpr.org/post/conn-c) | https://www.newhavenindepende | https://newhaven-ct.legistar.com/View.ashx? |
| --- | --- | --- | --- | --- | --- | --- | --- | --- | --- | --- | --- | --- | --- | --- | --- | --- | --- | --- | --- | --- | --- | --- | --- | --- | --- |
| CT | New London | 7/20/2020 | 7/20/2020 | passed by 7-0 vote; | City Council (under  president Efraín Domínguez, Jr.) | N |  | N |  | N |  | N |  | N |  | Defining racism and its  impact/consequences on minorities; Action Steps | systemic racism interpersonal racism health outcomes  criminal justice system and prison industrial complex SDOH  centering community experiences and voices | identify specific activities to ensure antiracism principles across leadership, staffing and contracting; Promoting equity through policies and budget; address and dismantle racism's effects on human/social services, education, economic development and public safety; improve data collection; partner with other organization confronting racism; improving communication and  community engagement; identify clear goals, objectives, and systems | N |  |  |  | <http://www.ci.new-london.ct.us/fil> | <http://www.ci.new-london.ct.us/fil> | <http://www.ci.new-london.ct.us/filestorage/4> |
| CT | Simpbury | 9/29/2020 | 9/29/2020 | Idea presented in June 2020 meeting; passed Unanimously | Board of Selectmen | N |  | N |  | N |  | Y | People of color in Connecticut  bear the burden of infant mortality | N |  | Defining racism along with  consequences for people of Color in the state and City; Action Steps | interpersonal racism systemic racism  criminal justice system and prison industrial complex health outcomes healthcare access  COVID-19 | enhance diversity to progress equity and justice oriented organization; promote equity through all policies to dismantle systemic racism; advocate locally for relevant policies; solidify alliances and partnerships with other organisms fighting racism;  support community efforts to amplify issues of racism; identify clear goals and periodic reports | N |  |  |  | https://[www.courant.com/commu](http://www.courant.com/commu) |  | https://[www.simsbury-ct.gov/sites/g/files/vyh](http://www.simsbury-ct.gov/sites/g/files/vyh) |
| CT | South Windsor | 6/15/2020 | n/a | First seen in 6/15/20 agenda; seen again in 9/9/20 agenda as a topic for discussion | Councilor Evans | N |  | N |  | N |  | N |  | N |  | Describes the need to address systemic racism  Describes Sub-Committee and its  actions | Black Lives Matter systemic racism centering community experiences and voices  COVID-19 | Establish Community Conversation around Black Lives Matter  Draft Statement of Support regarding Black Lives  Matter | N |  |  | Resolution is for the creation of Sub-Committee to Address Black Lives Matter Movement in the  town | https://[www.southwindsor-ct.gov/](http://www.southwindsor-ct.gov/) |  | https://[www.southwindsor-ct.gov/sites/g/file](http://www.southwindsor-ct.gov/sites/g/file) |
| CT | West Hartford |  | 6/23/2020 | Unanimous vote | Mayor Shari Cantor put forth the motion to adopt. | N |  | N |  | N | *For the CT ones I reviewed, nothing unique, but broad statements included ... see note on  infant mortality." JLS | Y | people of color in Connecticut bearing a disproportionate burden of illness and mortality including COVID-19 infection and death, heart disease, diabetes,  and infant mortality; | N |  | Describes manifestations of racism- poverty, health outcomes, access to care, etc.  Vague actions | health outcomes racial disparities COVID-19  SDOH | "Similar language to other cities in CT.  .... will work to progress as an equity and justice- oriented organization, by continuing to identify specific activities to enhance diversity and to ensure antiracism principles across our leadership, staffing and contracting language present in  other CT  Enhance educational efforts aimed at understanding and dismantling racism  Improve quality of data collected same as other cities in this state." | N |  | None |  | https://go.boarddocs.com/ct/west | h | https://go.boarddocs.com/ct/westh/Board.ns |
| CT | Windham | 7/6/2020 (on agenda) | 7/7/2020 | not found | Councilmember Nile | N |  | N |  | N |  | Y | people of color in the state experience disproportionate burden of infant mortality | N |  | Defining Racism and its consequences; Examples of health disparities; Actions steps | systemic racism interpersonal racism SDOH  COVID-19  healthcare access health outcomes | enhance diversity to progress equity and justice oriented organization; promote equity through all policies; improve quality and analysis of data that the Town collects; advocate for policies (local, state, regional, federal) to dismantle systemic racism; partner with other organizations confronting racism; support community efforts for  the issue of racism; periodic reports to city council | N |  |  |  |  |  | https://[www.windhamct.com/resources/minu](http://www.windhamct.com/resources/minu) |
| CT | Windsor, CT | 6/15/2020 | 6/15/2020 |  | Councilor Nuchette Black- Burke | N |  | N | broad statement that people of color in CT have a disproportionate burden of COVID-19, heart disease, diabetes and infant mortality | N |  | Y | broad mention that people of  color in CT have higher rates of infant mortality | N |  | Identify specific practices to enhance diversity and ensure antiracism principles across the organization.  Collect better data |  | enhance diversity to progress equity and justice oriented organization; promote equity through all policies; improve quality and analysis of data that the Town collects; advocate for policies (local, state, regional, federal) to dismantle systemic racism; partner with other organizations confronting racism; support community efforts for  the issue of racism; periodic reports to city council | N |  |  |  | https://townofwindsorct.com/app/ | https://[www.courant.com/news/co](http://www.courant.com/news/co) | https://townofwindsorct.com/app/uploads/m |
| FL | Hillsborough County | 9/10/2020 | 9/16/2020 | Board of county commissioners | Commissioner Pat Kemp | N |  | N |  | Y |  | Y | Black and Hispanic babies are more likely to die in their first  year of life | N |  | Define racism and consequences for communities of color; State and  county specific statistics; action steps | racial disparities interpersonal racism systemic racism  criminal justice system and prison industrial complex SDOH  racial justice COVID-19  health outcomes health equity  Black Lives Matter | enhance diversity to progress equity and justice oriented organization; promote equity through all policies to dismantle systemic racism; advocate locally for relevant policies; solidify alliances and partnerships with other organisms fighting racism; support community efforts to amplify issues of racism; promote and encourage racial equity  training; identify clear goals and periodic reports | N |  |  |  | https:// eagenda.hillsboroughcounty.org/ portal/PTL29560/search?  D=09/16/2020&T=Regular  %20BOCC  %20Meeting&Y=Backup&o=F- 2.pdf |  | https://eagenda.hillsboroughcounty.org/port |
| GA | DeKalb County | 6/15/2020 | 7/14/2020 | All Commission Districts Requested to Pass the resolution | Commissioner Larry Johnson | N |  | N |  | N |  | Y | African-American residents also have higher levels of infant  mortality and lower birth rates in the county | N |  | Defining Racism and its consequences; Examples of health disparities in the county; Actions steps | interpersonal racism systemic racism  criminal justice system and prison industrial complex SDOH  racial disparities health outcomes COVID-19 | enhance diversity to progress equity and justice oriented organization; promote equity through all policies; Racial Equity training; advocate locally and through the National Association of Counties; partner with other organizations confronting racism; support community efforts for the issue of  racism; periodic reports to the Governing Authority | N |  |  |  | https://[www.ajc.com/news/local/d](http://www.ajc.com/news/local/d) |  | https://[www.scribd.com/document/47003232](http://www.scribd.com/document/47003232) |
| IL | Cook County, IL | 6/27/2019 | 7/25/2019 | First proposed by Board of Commissioners 6/27/19  Referred to Health & Hospitals Committee 6/27/19  Modified- recommended for approval as substituted and accepted as substituted by Health & Hospital Committee 7/24/19 Approved by Board of Commissioners 7/25/19 | Commissioner Dennis Deer | N |  | N |  | N |  | Y | infant mortality rates in the county are double for African American Mothers (compared to Hispanic and Non-Hispanic White  counterparts) | N |  | Defining Racism, Background/Demographics of County, Statistics of Health Disparities, Action Steps (related to organizations and  community partners) | health outcomes health equity SDOH  interpersonal racism systemic racism racial disparities centering community  experiences and voices racial justice | Create the Office of Health and Social Equity; engage all citizens in racial justice work; recommend solutions to overcome racism; assess internal policy and procedures; create an inclusive organization in county; encourage other local, State and national entities to recognize racism as a public health crisis | N |  |  |  |  |  | https://cook-county.legistar.com/LegislationD |
| IN | Evansville, Indiana | 6/17/2020 | 6/22/2020 | Introduced by city council, presented to Mayor Windhorst | Councilmembers Moore- Morley and Trockman | N |  | N |  | N |  | N |  | N |  | Defining Racism (citing COVID-19 Data), Declaring Racism as a public  health crisis and commitment to open discussion and analysis, Action Steps | COVID-19  racial disparities centering community experiences and voices systemic racism interpersonal racism  transparency to the community racial disparities  criminal justice system and prison industrial complex | Investigate disadvantaged neighborhoods; urge all city elected officials too review policies to eradicate implicit and explicit racial bias and develop racially equitable ones; utilize available tools to limit disparities, collect data regarding racial disparities | N |  |  |  |  |  | https://evansville.granicus.com/MetaViewer. |
| IN | Indianapolis, Marion County, Indiana | 6/8/2020 | 6/10/2020 | On 02/20/20 a proposal was adopted by the council unanimously declaring racism unjust  Proposed and adopted on 06/08/2020 by  unanimous voice vote to declare racism as a public health crisis | SaRita Hughes | Y | Segregation and linking school funding to property value in the past has led to racial disparities in educational attainment in  the county | N |  | Y |  | Y | higher rates of infant mortality due to living in geographic areas with less healthcare access as a  result of redlining | Y | states that unequal access to a  living wage is one impact of racism and its link to health | Defines terms related to racism and inequity; cites finding of national organizations relating to negative health outcomes and racism; Cites the negative impact of historical racism; COVID-19 data for black residents of  Marion County; Declaring public health crisis and action steps | health equity SDOH  healthcare access hate crimes health outcomes COVID-19  systemic racism interpersonal racism racial disparities redlining | Commitment to frank and open discussion of race and its impact of decisions; urge all city elected officials too review policies to eradicate implicit and explicit racial bias and develop racially equitable ones; utilize available tools to limit disparities, collect data regarding racial disparities |  |  | APHA, the American Academy of Pediatrics, the US Office of Disease Prevention, American College of Physicians, NIH |  |  |  | https://[www.indy.gov/api/v1/indy_proposal_](http://www.indy.gov/api/v1/indy_proposal_) |

| ME | Portland |  | 7/13/2020 | not found | Mayor Kate Synder | N |  | Y |  | Y |  | N |  | N |  | History of racism in the US and Maine; recognizing the fight for racial justice in the US today; defining racism and it's  consequences; actions | systemic racism Black Lives Matter police violence COVID-19  criminal justice system and prison industrial complex  racial disparities | establish Racial Equity Steering Committee to respond to systemic racism; Community conversation serries executed by appointed councilmembers; have the Portland Arts Committee respond to community offers of public  art to support racial equity (mural, abber, etc.) | N |  | World Health Organization; United Stated Office of  Disease Prevention | Mentioning of a future "Vision Statement" for the community by the Racial Equity Steering Committee to address institutional  Racism | https://civicclerk.blob.core.window |  | https://portlandme.civicclerk.com/Web/GenF |
| --- | --- | --- | --- | --- | --- | --- | --- | --- | --- | --- | --- | --- | --- | --- | --- | --- | --- | --- | --- | --- | --- | --- | --- | --- | --- |
| MA | Beverly | 8/24/2020 | 8/24/2020 | Executive Order By Mayor | Mayor Michael Cahill | N |  | N |  | N |  | N |  | N |  | Defining racism along with present consequences for people of color;  Action Steps | SDOH  health equity racial justice COVID-19  systemic racism hate crimes police violence  centering community  experiences and voices | Engage historically marginalized Communities; Review policies and practices and implement needed changes; Develop an equity plan with objectives; Collect, make available, and analyze race and ethnic data; focus on access to prevention and treatment that is culturally and linguistically competent; work with existing organizations;  advocate for change in state and federal level | Y | Call for City Departments to allocate funding to implement  action steps |  |  | https://[www.beverlyma.gov/Docu](http://www.beverlyma.gov/Docu) |  | https://[www.beverlyma.gov/DocumentCente](http://www.beverlyma.gov/DocumentCente) |
| MA | Boston, MA |  | 6/12/2020 | An Executive Order  by the Mayor of Boston | Mayor Martin Walsh | N |  | N |  | N |  | N |  | N |  | Defining Racism Boston’s History of Racial Justice Work  Impact of COVID-19  8 Key Strategies Section | racial justice SDOH  healthcare access health equity health outcomes COVID-19  task force systemic racism | dismantle systemic racism, partner with city’s COVID-19 Task Force, engage with historically marginalized communities, access and analyze specific race and ethnic data to understand factors contributing to racism, focus of access to prevention and treatment | Y | funding to be applied consistent with executive order |  | Eight Key Strategies of the Boston Public Health Commission | https://[www.npr.org/sections/live-](http://www.npr.org/sections/live-) |  | https://[www.boston.gov/sites/default/files/fil](http://www.boston.gov/sites/default/files/fil) |
| MA | Chicopee, MA |  | 6/16/2020 | On agenda of City Council Meeting 06/16/2020,  motion passed through all stages by unanimous roll call vote 13-0 | Councilor Joel McAuliffe | N |  | N |  | N |  | N |  | N |  | n/a | COVID-19  systemic racism Black Lives Matter police reform | Mentions police department change and creating a culture of trust | N |  |  | Language lifted form Springfield, MA resolution  No clear actions that can be implemented by city council | <http://chicopeema.gov/AgendaCen> | https://[www.westernmassnews.co](http://www.westernmassnews.co/) | <http://chicopeema.gov/AgendaCenter/ViewFi> |
| MA | Everette, MA |  | 6/16/2020 | City Council makes resolution and refers the Mayor’s office to declare racism a public health crisis; Mayor made declaration of racism as  public health crisis 06/16/2020 | Councilor Gerly Adrien Mayor Carlo DeMaria | N |  | N |  | N |  | N |  | N |  | n/a | health outcomes police reform  transparency to community centering community experiences and voices | review and reform police use of force policies (bar chokeholds); engage communities; report review findings to communities; Treatment, assessment, and financial investment to eradicate negative health impacts, Police Reform and reallocating  funding for mental health resources | Y | financial investment to eradicate negative health impacts, Police Reform and reallocating funding for mental  health resources |  |  | <http://www.ci.everett.ma.us/Agen> | d | Only Could Access Agenda:  <http://www.ci.everett.ma.us/AgendaCenter/> ViewFile/Agenda/_06082020-1585 |
| MA | Framingham, MA |  | 6/16/2020 | Joint order by Mayor Spicer and Framingham Board of Health | Mayor Spicer and City Board of Health | N |  | N |  | N |  | N |  | N |  | Defining Racism Impact of COVID-19  8 Key Strategies (mirrors Boston) | systemic racism racial justice SDOH  health access health equity health outcomes COVID-19 | dismantle systemic racism, partner with city’s COVID-19 Task Force, engage with historically marginalized communities, access and analyze specific race and ethnic data to understand factors contributing to racism, focus of access to prevention and treatment | N |  |  | Mirrors Boston Executive Order |  |  | https://[www.framinghamma.gov/DocumentC](http://www.framinghamma.gov/DocumentC) |
| MA | Holyoke, MA | 6/17/2020 | 6/17/2020 | Executive Order by Mayor | Mayor Alex Morse | N |  | N |  | Y |  | N |  | N |  | Defining Racism and Examples of its Impacts including related to police violence; Declare Racism and police violence as a public health emergency; Five detailed action steps; Orders funding and other resources by every City cabinet, department, agency, and  office | police violence SDOH  healthcare access COVID-19  health outcomes racial justice systemic racism  interpersonal racism | dismantle Systemic Racism through: A) Creation of a Racial Equity Public Health Professional position within Board of Health B) Establish Citizen Police Advisory Committee to the Mayor C) The recognition of “Juneteenth Independence Day,” as a paid day off for City employees D) Support through advocacy and funding at the state and federal level for policies and funding opportunities  combating systemic racism | N |  |  |  |  |  | https://docs.google.com/viewerng/viewer?url |
| MA | Longmeadow | 7/6/2020 | 7/20/2020 | Brought forth at 7/6/20 meeting and unanimously approved by vote on 7/20/20  meeting | Board Member Richard Foster and Chair Thomas  Lachiusa | N |  | N |  | N |  | N |  | N |  | Defining racism along with it's implications and impacts for  communities of color; action steps | systemic racism interpersonal racism health outcomes healthcare access police violence Black Lives Matter racial justice  health equity centering community experiences and voices  police reform | establish a working group to confront systemic (through policies, citizen engagement, hiring process, partnering with organizations)  Police Reform  Increase access to opportunity Listen to unheard community voices | N |  | American Academy of Pediatrics  American Medical Association  American Public Health  Association |  | https://[www.thereminder.com/](http://www.thereminder.com/) localnews/longmeadow/board- declares-racism-public-health-  crisis-changes/ | https://[www.masslive.com/news/](http://www.masslive.com/news/) 2020/09/longmeadow-select- board-to-form-coalition-for-racial-  justice.html | https://[www.longmeadow.org/](http://www.longmeadow.org/) AgendaCenter/ViewFile/Item/742?  fileID=17645 |
| MA | Medford, MA | 6/9/2020 | 6/15/2020 | Resolution offered by Councilor Bears and Morell and invited to be joint resolution with Mayor and city school committee during City Council meeting (6/9/2020); moved for approval (four in favor, three opposed on roll  call vote); Officially declared on 6/9/2020 by Mayor and city Board of Health | Councilor Bears and  Morell w/ Mayor Breanna Lungo-Koehn | N |  | N |  | N |  | N |  | N |  | definition and examples of racism and social determinants of health (COVID19 cited) and their effects; Commitment to address change and  declaration of racism as public health crisis; actions steps | SDOH  health equity healthcare access health outcome COVID-19  systemic racism police reform | create policies to address institutional racism along with existing policies and work around health, engage historically underserved and marginalized groups, Enhance the city’s hiring to promote equity | N |  |  |  | https://library.municode.com/ma/ |  | <http://www.medfordma.org/storage/2020/06> |
| MA | Revere | 6/25/2020 | 6/25/2020 | Mayor Arrigo made declaration with Revere  Board of Health and Director of Healthy Community Initiatives | Mayor Brian Arrigo | N |  | N |  | N |  | N |  | N |  | Recognizes the need to address systemic racism and its consequences; Highlights the work the city has already done in terms of racial justice  and how it will expand on this | SDOH COVID-19  health outcomes racial disparities racial justice  systemic racism | reinstatement of the City’s Human Rights  Commission and Appointment of its Executive Director; | Y? | mention of declaring paving the  way for funding to address SDOH and racial equity | Revere Human Rights Commission |  |  |  | Only able to access press release:  https://[www.revere.org/news/post/mayor-](http://www.revere.org/news/post/mayor-)  arrigo-and-revere-board-of-health-declare- racism-a-public-health-crisis |
| MA | Somerville, MA | 6/11/2020 | 8/27/2020 | Declared 06/11, laid on table by city council until 08/27, approved by city council by unanimous vote but sent for follow up by  Public Health and Public Safety committee | Mayor Curtatone, the Chief of Police and the Director of HHS; Councilor  Ballantyne | N |  | N |  | N |  | N |  | N |  | Declaration by Mayor, the Chief of Police and the Director of HHS of Systemic Racism as a Public Safety and Health Emergency; 10 point plan (via press release); specifics on police  reform | systemic racism police reform | Proposed 2021 budget will include the hiring of independent facilitator for a civilian oversight committee of the city’s police department, resolution in support of body cameras, 10 point plan related to police and community support in terms of racism as a public health crisis | Y | Proposed 2021 budget will include police reform  consideration |  |  | <http://somervillecityma.iqm2.com/> | <http://somervillecityma.iqm2.com/> | Only Could Access Meeting Minutes: <http://somervillecityma.iqm2.com/Citizens/> FileOpen.aspx?  Type=15&ID=2505&Inline=True |
| MA | Springfield, MA |  | 6/22/2020 | Resolution by city council (with Mayor) | Initiated by Councilor Adam Gomez joined by  Mayor Sarno | N |  | N |  | N |  | Y | higher levels of infant mortality and lower birth weights for African Americans used as one of many examples of racism and segregation causing a health  divide in the state | N |  | Defining racism w/ examples (statewide and nationally), Action  Steps | SDOH  systemic racism interpersonal racism racial disparities  criminal justice system and prison industrial complex COVID-19  health outcomes healthcare access centering community  experiences and voices | review of police (strengthen Community Police Hearing Board); Establish Office of Racial Equity in Springfield DHHS; promote racially equitable policies and community support | N |  |  |  | https://springfieldcityma.iqm2.com | https://springfieldcityma.iqm2.com | https://springfieldcityma.iqm2.com/Citizens/D |
| MD | Anne Arundel County |  | 11/1/2019 | Proposed by former councilman Smith in 2017 but lacked need votes to pass, declared by  County Executive Steuart Pittman and Health Officer Nilesh Kalyanaraman | Steuart Pittman and Nilesh | N |  | N |  | Y |  | N |  | N |  | n/a | healthcare access health outcomes | New Office of Health Equity and Racial Justice was created | N |  |  |  | https://[www.aacounty.org/boards-](http://www.aacounty.org/boards-) |  | https://[www.aacounty.org/boards-and-](http://www.aacounty.org/boards-and-)  commissions/human-relations-commission- of-anne-arundel-county/ |
| MD | Montgomery County | 6/9/2020 | 6/16/2020 | n/a | Councilmember Will Jawando | Y |  | N |  | N |  | Y | Racism and de facto segregation exacerbate health divide resulting in African Americans having higher levels of infant and maternal mortality as well as  higher levels of low birth weight | N |  | Background of racism in the county  and its response to racism, detailed/itemized action section | COVID-19  health outcomes centering community experiences and voices racial justice  systemic racism interpersonal racism criminal justice system and  prison industrial complex | create new policies to lessen the impact of racism, become an equity and justice-oriented organization, collaborating with local communities, encourage racial equity and social justice training | N |  |  |  |  |  | https://[www.montgomerycountymd.gov/cou](http://www.montgomerycountymd.gov/cou) |
| MD | Prince George County | 7/14/2020 | 7/21/2020 | City Council Introduced resolution and referred to Committee of the Whole (COW) 7/14/20; COW favorably recommended 7/15/20; City Council Amended resolution;  Voted and adopted 11-0 7/21/20 | Sydney J. Harrison and Calvin S. Hawkin | Y | The county had the largest population of enslaved persons in Maryland; schools were not integrated until 1972 (18 years after Brown v. Board of  Education) | N |  | N |  | N |  | N |  | defining racism, and its history in the county; Consequences of racism to black and Latino communities in the  county; Action Steps | systemic racism police violence SDOH  hate crimes task force health equity  criminal justice system and prison industrial complex | systemic reforms of equity; establish racial equity task force to deactivate systemic racism and establish equity for health; create policies and legislation for equitable communities; support policies that avoid criminalization of substances and mental illness; trauma informed policies;  police reform (related to training i.e. use-of-force, chokeholds, de-escalation) | N |  |  |  |  |  | https://princegeorgescountymd.legistar.com/ |
| MI | Eaton County | 6/17/2020 | 6/17/2020 | Moved by Brehler on at regular meeting and approved the same day | Commissioner Brehler | Y |  | Y |  | Y |  | Y | health disparities result in higher rates of infant mortality for  African Americans in Michigan | N |  | Defines Racism and example of its consequences  History of Racism (at national, state, and county/city level)  Action Steps | systemic racism interpersonal racism redlining  criminal justice system and prison industrial complex healthcare access  COVID-19  centering community experiences and voices | advocate for relevant policies to improve health in black community;  support local, state, and federal initiatives that advance social justice;  assess local ordinances, health regulations and internal policies and procedures to promote diverse discission making;  urge other governmental bodies to combat  systemic racism | N |  | American Public Health Association;  National Association of County and City Health Officials; American Academy  of Pediatrics |  |  |  | https://[www.eatoncounty.org/ArchiveCenter/](http://www.eatoncounty.org/ArchiveCenter/) |
| MI | Flint, MI |  | 6/1/2020 | Mayor created Black Lives Matter Advisory Council for Flint Police Department, resulted in  City Council considering and passing resolution | Mayor Meely, City Council President Monica  Galloway | Y |  | N |  | N |  | N |  | N |  | defining racism, state specific statistics of the impacts of racism and COVID-  19, City Council’s Action Step | Black Lives Matter systemic racism SDOH  criminal justice system and prison industrial complex COVID-19  health outcomes | creation of Black Lives Matter Advisory Council, draft of new ordinance forbidding bias crime reporting, denounce use of illegal restraint methods, progress antiracism principles across the  City | N |  |  |  | <http://www.mml.org/wp-content/u> |  | <http://www.mml.org/wp-content/uploads/20> |
| MI | Genesee County, MI | 6/3/2020 | 6/10/2020 | Resolution was presented to county board of health by group of concerned citizens, board of health voted unanimously to adopt, presented to Board of Commissioners for approval | Commissioner Bryant Nolden | N |  | N |  | N |  | Y | infant mortality rate for infants of non-Hispanic black women is  almost twice that of white women | N |  | Defining aspects of racism and county data/policies, action steps, statement to consider attaching funds to action  steps | SDOH  health outcomes racial justice systemic racism interpersonal racism  criminal justice system and  prison industrial complex | Conduct an assessment of internal policy and procedures to ensure racial equity, create an equity and justice-oriented organization, incorporate educational efforts to address and dismantle racism, advocate for relevant policies that improve health in communities of color, work to build alliances and partnerships with other  organizations | Y | board will consider allocating adequate financial resources within its budget to accomplish these activities (organizations  not specified) |  |  | [http://geneseecountymi.iqm2.com](http://geneseecountymi.iqm2.com/) |  | <http://geneseecountymi.iqm2.com/Citizens/F> |

| MI | Ingham County, MI |  | 6/9/2020 | Approved after meeting with County Board of Commissioners | Commissioner Derrell Slaughter | Y |  | Y |  | Y |  | Y | Black Citizens have higher rates of infant and maternal mortality | N |  | Defining Racism, Historical Contexts of Racism, Present day Examples of  Racism in the US and the County, Action Steps | interpersonal racism systemic racism  criminal justice system and prison industrial complex police violence  health equity redlining COVID-19  centering community experiences and voices racial justice | advocate for relevant policies that improve health in the Black community and support racial justice, assess current and proposed laws and policies | N |  |  |  | https://ingham.org/NewsEvents/Ne |  | <http://www.hd.ingham.org/Portals/HD/Home> |
| --- | --- | --- | --- | --- | --- | --- | --- | --- | --- | --- | --- | --- | --- | --- | --- | --- | --- | --- | --- | --- | --- | --- | --- | --- | --- |
| MI | Jackson, MI | 6/16/2020 | 6/16/2020 | Resolution to declare racism a public health crisis failed 2-7, a counter Racial Equity Resolution condoning discrimination was  passed 7-1 | Commissioner Daniel Mahoney (racism as a public health crisis), Commissioner David Elwell (counter Racial Equity Resolution condoning discrimination) | N |  | N |  | N |  | N |  | N |  | Counties Commitment to safe and welcoming community; acknowledge discrimination and bias due to physical  characteristics; action steps to address these biases | n/a | creation of an ad hoc committee of three  Commissioners and four County residents to develop anti-discrimination policy | N |  |  | Does not declare racism as a public health crisis. Original declaration rejected. Counter resolution passed, omitting talks of racism as a public health crisis. Talks about discrimination related  to "biases" of physical characteristics | https://[www.scribd.com/documen](http://www.scribd.com/documen) | https://[www.mlive.com/news/kala](http://www.mlive.com/news/kala) | https://mi-jackson.civicplus.com/AgendaCent |
| MI | Kalamazoo County, MI |  | 6/16/2020 | proclamation to declared racism as a public health crisis approved on 6/16 along with  proclamation to end police brutality | County Commissioner Stephanie Moore | Y |  | Y |  | Y |  | Y | black residents in the county have higher rates of infant and  maternal mortality | N |  | Defining racism and its history in the US, details on the consequences of racism in general and in the  state/county, action steps | interpersonal racism systemic racism redlining  COVID-19  health outcomes police violence task force  criminal justice system and prison industrial complex health equity  racial disparities | recommitting our full attention to improving the quality of life and health, advocate for relevant policies, assess our current and proposed laws (ordinances and health regulations) and policies, and their implementation, assess internal policies and procedures to ensure racial equity, expand the  role of the Equity Taskforce | N |  |  |  | https://wwmt.com/news/local/kala |  | https://kalamazoocomi.civicclerk.com/Web/U |
| MI | Lansing, MI |  | 6/22/2020 | Mayor Andy Schor announced the city’s first concrete response to the ongoing social unrest in the form of a one-page document titled “Racial Justice and Equity Community Action Proposals,” followed by this resolution by the  City Council | Commissioner Derrell Slaughter | Y |  | Y |  | Y |  | Y | black residents have higher rates of infant and maternal mortality | N |  | Defining racism and its history in the US, details on the consequences of racism in general and in the  state/county, action steps | **interpersonal racism systemic racism redlining**  **COVID-19**  **police violence criminal justice system and prison industrial complex**  **healthcare access** | calls for the establishment of a standing Committee on Equity, Diversity and Inclusion designed to establish policies that can improve life for the local Black community, Lansing City Council will establish a standing committee on Equity, Diversity, and Inclusion to establish relevant  policies | Y | proposal from the mayor to create an “Equity and Anti- Discrimination Fund” using  $100,000 from Police Department budget, $20,000 from the Mayor’s Office and  $50,000 from the Human Relations and Community Services office |  |  | https://[www.lansingcitypulse.com/](http://www.lansingcitypulse.com/) |  | https://[www.lansingmi.gov/DocumentCenter](http://www.lansingmi.gov/DocumentCenter) |
| MI | Pontiac | 6/9/2020 | 6/9/2020 | not found | City Council President Kermit Williams | N |  | N |  | N |  | Y | In Michigan, the highest excess death rates exist for African Americans for infant mortality, maternal mortality, and pediatric  asthma | N |  | Defines Racism and its consequences (specifically in the State)  Action Steps | interpersonal racism systemic racism SDOH  health outcomes task force  COVID-19  racial justice criminal justice health equity | Conduct an assessment of internal policy/procedures  Create an equity and justice oriented organization Increase diversity and incorporate anti-racism principles  Incorporate educational efforts Advocate for relevant policies and build  alliances/partnerships with other organizations fighting racism | N |  | Michigan Coronavirus Task Force on Racial Disparities | Mentions the United Nations declaration of a decade (2015-  2014) focused on the people of African descent | https:// [www.theoaklandpress.com/news/](http://www.theoaklandpress.com/news/) local/pontiac-city-council- declares-racism-a-public-health- crisis-vowing-internal-review/ article_9247c8c6-ab45-11ea-  8d85-93c2eb2abf06.html |  | <http://www.pontiac.mi.us/councilagendapack> |
| MI | Port Huron, MI |  | 6/8/2020 | Councilwoman Ashford requested after discussing with community leaders | Councilmember Anita Ashford | N |  | N |  | N |  | N |  | N |  | Defining racism and its impact, details of racism in the city consequences of  and COVID-19, action steps | systemic racism interpersonal racism SDOH  COVID-19  health outcomes | Work to progress as an equity and justice-oriented organization, Promote equity through all policies approved, continue to advocate locally, partner with other organizations confronting racism, support community efforts, continue racial equity training, identify clear goals and report to City Council to assess progress | N |  |  |  | https://[www.thetimesherald.com/s](http://www.thetimesherald.com/s) |  | <http://porthuroncitymi.iqm2.com/Citizens/Fil> |
| MI | Washtenaw County, MI | 6/30/2020 | 7/1/2020 | County passed resolution the day after the Board of health did | Commissioners Brabec, Jefferson and Scott | Y |  | N |  | Y |  | Y | devastating inequity with regard to infant mortality in the black  community | N |  | Defining racism and its impact, details of racism in the city consequences of  and COVID-19, action steps | systemic racism interpersonal racism healthcare access  criminal justice system and prison industrial complex health equity  SDOH COVID-19 | increase the budget for public health department and racial equity office, review county budget through a racial equity frame, utilize community- based budget, enact universal paid leave for employees, increase representation in Community Advisory Board for Law Enforcement, review sheriff’s office’s use of force policies and endure their anti-racist approach to public safety | Y | increase the budget for public health department |  |  | https://[www.mlive.com/news/ann-](http://www.mlive.com/news/ann-) |  | https://[www.washtenaw.org/DocumentCente](http://www.washtenaw.org/DocumentCente) |
| MI | Wayne County |  | 7/2/2020 | Voted to approve 14-0, with all 14 commissioners cosponsoring resolution | Commissioner Alisha Bell | N |  | N |  | Y | Tuskegee Syphilis Study | N |  | N |  | N/A | healthcare access SDOH | talks of possible formation of committee  established by Governor Whitmer to look at data policies and eliminates forms of racism | N |  | Franklin County Ohio's Declaration | Franklin County Ohio's Declaration  (the first of this kind in 2020) inspired this declaration | https://[www.youtube.com/watch?](http://www.youtube.com/watch) | (YouTube link 5:40 to 19:17) | Only able to access news articles and Meeing Recording:  https://[www.youtube.com/watch?](http://www.youtube.com/watch) v=eh_OJVjUQtM https://[www.detroitnews.com/story/news/](http://www.detroitnews.com/story/news/) local/wayne-county/2020/07/02/wayne- county-commission-decries-racism-public-  health-crisis/5363697002/ |
| MI | Westland | 8/3/2020 | 8/3/2020 | Unanimously accepted 7-0 | Councilmember James Godbout | N |  | N |  | N |  | Y | In Michigan the highest excess death rates exist for African Americans for infant mortality, maternal mortality, and pediatric  asthma | N |  | Defining racism along with its impacts on communities of color; action steps | interpersonal racism systemic racism  criminal justice system and prison industrial complex SDOH  health outcomes task force  COVID-19  racial justice | Direct the Mayor to form a commission to implement this resolution; conduct an assessment of internal policy and procedures and also create an equity and justice and diversity oriented organization; identify specific activities to increase diversity and incorporate anti-racism principles; incorporate education efforts; advocate for relevant policies; support local, state, and federal initiatives; build alliances and partnerships with  other organizations | N |  |  | Mentions the United Nations declaration of a decade (2015- 2014) focused on the people of  African descent |  |  | https://mi-westland.civicplus.com/Document |
| MI | Ypsilanti, MI |  | 6/2/2020 | n/a | Mayor Pro Tem Lois Richardson | N |  | N |  | Y |  | N |  | N |  | acknowledges recent victims of police brutality resulting in civil unrest (Murders of Floyd, Arbery, Taylor), defining racism and its consequences  to African Americans | systemic racism interpersonal racism healthcare access  criminal justice system and prison industrial complex SDOH  health outcomes police violence | Work to progress as an equity and justice-oriented organization, Promote equity through all policies approved, continue to advocate locally, partner with other organizations confronting racism, support community efforts, continue racial equity training, identify clear goals and report to City Council to assess progress | N |  |  |  | https://cityofypsilanti.com/Docum | https://cityofypsilanti.com/Agenda | https://cityofypsilanti.com/AgendaCenter/Vie |
| MI | State At Large | 8/5/2020 | 8/5/2020 | Executive Directive by Governor | Governor Gretchen Whitmer | N |  | Y |  | Y |  | Y | People of color in the state  experience higher rates of infant and maternal mortality | N |  | Defining racism along with historical and present consequences for people of Color in the state; response of national health organizations and the current State Administrations; Action  Steps | criminal justice system and  prison industrial complex redlining  SDOH  racial justice task Force  racial disparities health equity centering community experiences and voices health outcomes COVID-19  hate Crimes police violence | Strategies related to data analysis, policy &  planning, engagement of community & advocacy, and training | Y | Call for State Departments to  allocate funding to implement action steps | APHA AMA  American Academy of Pediatrics  American College of Emergency Physicians | A Black Leadership Advisory Council created to advise Mayor (black led from various fields and  industries) |  |  | https://content.govdelivery.com/attachments |
| MN | Hennepin County | 6/23/2020 | 6/30/2020 | Board approved resolution 6-1  Further Debate resulted in a 4-3 vote to add 10 directives | Angela Conley and Irene Fernando (the board’s first two commissioners of  color) | N |  | N |  | Y |  | N |  | N |  | The county's mission, overview of public health definitions and goals; acknowledging the health disparities in the county in general and in regards to COVID-19; past research on health  outcomes and racism and noting other US counties making declarations | healthcare access health equity COVID-19  centering community experiences and voices systemic racism  health outcomes | 10 directives related to recognizing health disparities, improving data collection, policy and human resources assessment, reporting  mechanisms | Y | Incorporate racism and its  public health crisis into the 2021 budget meeting material |  |  | https://hennepin.novusagenda.co | mhttps://[www.startribune.com/henn](http://www.startribune.com/henn) | https://hennepin.novusagenda.com/agendap |
| MN | Minneapolis | 7/9/2020 | 7/17/2020 | approved by Policy & Government Oversight Committee on 7/9/20 ; approved by city council 7/17/20; approved and signed by mayor  7/20/20; published 7/25/20 | Councilmembers Andrea Jenkins (primary) and  Phillipe Cummingham | Y | acknowledges Minneapolis' physical placement on  Indigenous land | N |  | Y |  | Y | high infant and maternal mortality rates is an example of inequitable health outcomes for  BIPOC | N |  | recognizing racism in the city and the role that history plays; Consequences of racism in all areas of life; Cities stance, past actions, and proposed  actions | racial disparities redlining systemic racism  interpersonal racism criminal justice system and prison industrial complex COVD-19  health outcomes police violence centering community  experiences and voices | build active anti-racist culture; center community voices; prioritizes racial equity through policies; criminal justice reform; restore and increase the availability of high-quality youth development programming for BIPOC youth and young adults; implement an annual report with racially disaggregated data on the health of Minneapolis BIPOC; Build a workplace culture that promotes  racialized repair and cross-cultural relationships | Y | allocate in the budget advancing racial equity and reporting these results annually; Allocate dollars in the Mayor’s budget towards small business,  housing, community-based  infrastructure | APHA  American Medical Association  American Academy of  Pediatrics | City of Minneapolis’ Strategic and Racial Equity Action Plan aka SREAP (adopted in July 2019) 3 unique policy areas to enhance BIPOC communities by eliminating violence, reduce rental housing displacement, increase BIPOC  businesses ownership | https://lims.minneapolismn.gov/D | <http://news.minneapolismn.gov/> 2020/07/17/city-council-declares- racism-a-public-health-  emergency/ | https://lims.minneapolismn.gov/Download/R |
| MN | Olmsted County | 8/4/2020 | 8/4/2020 | Unanimously accepted | Commissioner Jim Bier | N |  | N |  | N |  | N |  | N |  | Defining racism along with its impacts on communities of color; action steps | COVID-19  health equity healthcare access health outcomes racial disparities | Study and investigate this issue with emphasis on services | N |  | APHA |  | [http://olmstedcountymn.iqm2.com](http://olmstedcountymn.iqm2.com/) |  | <http://olmstedcountymn.iqm2.com/Citizens/F> |
| MN | State At Large | 7/11/2020 | 7/20/2020 | put forward during the July special session; won by 82-40 | Rep. Ruth Richardson (DFL-Mendota Heights) | N |  | N |  | Y |  | N |  | N |  | defining racism, and its history; Consequences of racism to black and  indigenous communities in Minnesota | systemic racism interpersonal racism SDOH  health equity health outcomes | collaborate with state's law and agencies and justices agencies for equitable public safety; assessment of existing policies and practices through an intersectional lens of racial equity; human resources assessment; enhancing-data driven education; support local/regional/federal initiatives to dismantle systemic racism; convene a house select committee for the states response to  addressing racism; data driven education | N |  | APHA  American Medical Association  American Academy of Pediatrics  American College of Obstetrics and Gynecologists | Mentions employing an intersectional lens to racism  (LGBTQ+, immigrant, disabled, gender, etc.) | https://[www.revisor.mn.gov/bills/t](http://www.revisor.mn.gov/bills/t) |  | https://[www.revisor.mn.gov/bills/text.php?n](http://www.revisor.mn.gov/bills/text.php?n) |
| MO | Kansas City | 8/15/2019 | 8/28/2019 | Introduced by city council 8/15/19, referred to neighborhood planning and development  committee, on agenda 8/21/19 for 8/28/21 meeting, adopted unanimously 5-0 on 8/28/19 | Councilmember Robinson | N |  | N |  | Y |  | N |  | N |  | Defining racism and its consequences  Statistics on racial disparities in city Action Steps | systemic racism Interpersonal racism criminal justice system and prison industrial complex Health equity  Health outcomes SDOH  centering community experiences and voices Racial disparities  racial justice | Conduct an assessment of the city's internal policies to address public health disparities and racial inequities; create inclusive and diverse community; advocate for relevant policies;  encourage other local and state and national entities to do the same | N |  |  |  |  |  | <http://cityclerk.kcmo.org/liveweb/> Documents/Document.aspx? q=G43IjqpcYIFy5Mc5eD8WL  %2FBqWZF8MUCiAfJhOAeahnn7izONYV29% 2FhWjDQCHRyTu |

| NV | State At Large | 8/5/2020 | 8/5/2020 | Executive Order by Governor | Governor Steve Sisolak | N |  | N |  | N |  | N |  | N |  | Defining racism along with its impacts on state residents | COVID-19  racial justice health outcomes systemic racism racial disparities  criminal justice system and prison industrial complex SDOH  healthcare access | N/A | N |  |  |  | https://gov.nv.gov/News/Proclama |  | https://gov.nv.gov/News/Proclamations/2020 |
| --- | --- | --- | --- | --- | --- | --- | --- | --- | --- | --- | --- | --- | --- | --- | --- | --- | --- | --- | --- | --- | --- | --- | --- | --- | --- |
| NJ | Leonia | 6/1/2020 | 6/1/2020 | Brought forth by Mayor Zeigler, motioned by Councilwomen Davis, approved by council | Mayor Zeigler, Councilwomen Davis | N |  | N |  | N |  | N |  | N |  | Defines Racism and its consequences Action Steps | systemic racism interpersonal racism racial disparities SDOH  health outcomes  healthcare access COVID-19 | Promote/advocate for policies, education, and training related to dismantling systemic racism at the local, state, regional, and federal level;  Support community efforts to amplify issues of racism and engage with communities of color | N |  |  |  | https://docs.google.com/gview? url=https%3A%2F  %2Fleonianj.granicus.com  %2FDocumentViewer.php%3Ffile  %3Dleonianj_ea2e52018f881acc1 b26bc9ac061dae3.pdf%26view  %3D1&embedded=true |  | https://docs.google.com/gview?url=https%3A |
| NC | Ashville, NC |  | 7/14/2020 | Read, approved and adopted 7/14/2020 | N/A | N |  | N |  | Y | Mention Black people disproportionately being segregated to Brown zones and other toxic  waste sites | Y | Mentions a history of involuntary sterilization | N |  | Provides historical context to racial disparity  Issues an apology to the Black community for racist practices  Encourages additional funding, data, discussions to explore effect of racism Encourages  other states and localities to pass  similar legislation | systemic racism racial disparities  racial justice | Apologies and will make amends with Black community for slavery, discrimination, and gentrification  Call on other legislators (federal and state) to do the same  Plans to establish a plan that will boost the economic mobility of Black people  Plans to establish the Community Reparations Commission (within the next year) to repair  damage from the systemic racism | N |  |  | This is more about reparations for Black people of Asheville than it is about a public health effort. Little  mention of health related topic |  |  | https://drive.google.com/file/d/1WKialVISWz |
| NC | Charlotte, NC |  | 6/17/2020 | A proclamation signed by the mayor | n/a | Y |  | N |  | Y |  | N |  | N |  | Provides historical context to racial disparity Encourages additional funding, data, discussions to explore effect of racism  Encourages other states and localities to pass similar legislation | racial equity health equity  criminal justice system and prison industrial complex SDOH  COVID-19 | Promote racial equity through Charlotte City Council policy  Reduce racial disparity as critical priority | N |  |  | Does not provide action items/deliverables for the city to work on Identical  to county proclamation passed the day before |  |  | https://[www.wbtv.com/2020/06/17/mayor-vi](http://www.wbtv.com/2020/06/17/mayor-vi) |
| NC | Durham |  | 6/8/2020 | n/a | n/a | n |  | N |  | N |  | Y | discusses disproportionate infant mortality rates | N |  | Provides historical context to racial disparity  Encourages Bias training  Review policies and programs for racial bias Advocate  and promote policies to help racism | systemic racism racial disparities health equity  COVID-19 | Support policies to promote racial equity Encourage bias training for commissioners and affiliated partners  Create periodic reviews to assess racial equity  progress | n |  |  |  |  |  | https://[www.dconc.gov/home/showdocumen](http://www.dconc.gov/home/showdocumen) |
| NC | Mecklenburg County |  | 6/16/2020 | A proclamation signed county commissioners | n/a | Y |  | N |  | Y |  | Y | “There is also evidence suggesting that the racism endured by black mothers contributes to the high  maternal and infant mortality rate” | N |  | Provides historical context to racial disparity Encourages additional funding, data, discussions to explore effect of racism  Encourages other states and localities to pass similar legislation | racial equity health equity  criminal justice system and prison industrial complex SDOH  health disparities COVID-19 | Promote racial equity through Mecklenburgh County Council policy  Reduce racial disparity as critical priority | n |  |  | Does not provide action  items/deliverables for the city to work on |  |  | <http://mecklenburg.legistar.com/gateway.asp> |
| NC | New Hanover County |  | 7/13/2020 | n/a | Chair Olson-Boseman | n |  | n |  | n |  | Y | discusses disproportionate infant mortality rates | N |  | Provides historical context to racial disparity Encourages additional funding, data, discussions to explore effect of racism  Encourages other states and localities to pass similar legislation | systemic racism racial disparities health equity  COVID-19 | Encourage others (state, cities, county) to promote racial equity policies  Encourages further research on the issue | N |  |  |  |  |  | https://laserfiche.nhcgov.com/weblink/0/doc |
| NC | Pitt County |  | 8/3/2020 | n/a | n/a | N |  | N |  | Y |  | Y | Discussed funding/hiring of infant/maternal researchers and  nurses | N |  | Provides historical context to racial disparity Encourages additional funding, data, discussions to explore effect of racism  Encourages other states and  localities to pass similar legislation | systemic racism racial disparities health equity  COVID-19 | Encourage others (state, cities, county) to promote racial equity policies  Encourages further research on the issue | N |  |  |  |  |  | https://[www.pittcountync.gov/AgendaCenter](http://www.pittcountync.gov/AgendaCenter) |
| NC | Wake County |  | 7/6/2020 | N/a | n/a | N |  | N |  | N |  | N |  | Y | Mentions county has living wage policy | Provides historical context to racial disparity Encourages additional funding, data, discussions to explore effect of racism  Encourages other states and localities to pass similar legislation | systemic racism racial disparities health equity  COVID-19 | Encourage others (state, cities, county) to promote racial equity policies  Encourages further research on the issue | N |  |  |  |  |  | <http://pulse.ncpolicywatch.org/wp-content/u> |
| OH | Akron, OH | 6/5/2020 | 6/8/2020 | Resolution submitted to the city council, pass 2/3rds vote by council | Mayor Horrigan President  Sommerville Councilwoman Samples | Y |  | N |  | Y |  | Y | Black children 3x more likely before 1st birthday | N |  | Historical context on racism Statistics on racial disparities  Develop 5 year equity and social justice plan Promote  equitable policies | racial equity SDOH  task force | To declare racism is a health crisis, and support  equitable policies and to inform our public discourse on racism. |  |  |  |  | https://[www.cleveland.com/akron/](http://www.cleveland.com/akron/) |  | https://onlinedocs.akronohio.gov/OnBaseAge |
| OH | Athens, OH |  | 6/22/2020 |  |  | N |  | Y |  | Y |  | Y | Mentions higher infant and  maternal mortality rates amongst Black people | N |  | Identify disparities and inequities Provide historical context  Seeks to support future efforts and resolutions for the issue | systemic racism interpersonal racism racial disparities SDOH | Ask Mayor to establish working group to address racial equity  Strengthen community relationships  Support efforts to mitigate racism Review city code with racial equity lens | N |  |  |  |  |  | Only Able to Access News Article:  https://[www.thepostathens.com/article/](http://www.thepostathens.com/article/)  2020/06/city-council-racism-public-health- crisis |
| OH | Canton, OH | 6/15/2020 | 6/22/2020 | Proposed to canton city council and approved by canton city board of health | Greg Hawk | N |  | Y |  | N |  | Y | Black infants have higher mortality and lower birth weights | N |  | Engage community stakeholders Bias training  Review policies and programs for  racial bias Advocate and promote policies to help racim | racial equity SDOH  health disparities COVID-19 | Establish Public Health and Race Equity committee  Systemic review of Canton City Public Health department programs  Bias training for committee and committee members | N |  |  |  | <http://cantonhealth.org/pdf/2020-> |  | Only Could Access Agenda:  https://[www.cantonohio.gov/](http://www.cantonohio.gov/)  AgendaCenter/ViewFile/Agenda/_06152020- 495 |

| OH | Cincinnati, OH | 7/30/2020 | 8/5/2020 | Proposed to council on 7/30/2020 Passed 8/5/2020 | Andrew W. Garth, Interim City Solicitor | y |  | N |  | N |  | Y | Black infants higher mortality rates  Black infants have the same life expectancy as white infants 50 years agp | N |  | Identify disparities and inequities Provide historical context  Seeks to support future efforts and resolutions for the issue | systemic racism interpersonal racism racial disparities  criminal justice system and prison industrial complex SDOH | Establish working group of community leaders and health professionals to address racial equity Strengthen community relationships  Support efforts to mitigate racism  Review city policies with racial equity lens | N |  | Cincinnati NAACP, the Urban League  of Southwestern Ohio, United Way of Greater Cincinnati, Cradle Cincinnati, the Center for  Department Board of Health, Cincinnati Children's Hospital, University of Cincinnati Medical  Center, the Cincinnati Board of Education, the YWCA, Hamilton County Board of Commissioners, and All-In  Cincinnati |  |  |  | https://cincinnatioh.legistar.com/LegislationD |
| --- | --- | --- | --- | --- | --- | --- | --- | --- | --- | --- | --- | --- | --- | --- | --- | --- | --- | --- | --- | --- | --- | --- | --- | --- | --- |
| OH | Cleveland, OH | 3/13/2020 | 6/3/2020 | Proposed to Cleveland city council Approved to Cleveland city council | Councilmembers: Blaine Griffin  Basheer Jones Kerry McCormack | Y |  | Y |  | Y |  | Y | 12x as many incarcerated black youth than whites  Black youth have more adverse childhood experiences  Ohio has one of the worst infant mortality rates  Cuyahoga county has the worst black infant mortality in Ohio | Y | Black residents have 2x as high poverty rates and lower median income | Identify disparities and inequities Provide historical context  Seeks to support future efforts and resolutions for the issue | health outcomes SDOH  systemic racism redlining  racial disparities  criminal justice system and prison industrial complex health outcomes | Supports establishment of working group for racial equity  Seek solutions for racial justice Build community relations  Promote racially equitable hiring and promotions Advocate and draft relevant health policies | N |  | WHO ODPHP  Boston Public Health Commission NAACP Cleveland Branch  Urban League of Greater Cleveland YWCA of Greater Cleveland  First Year Cleveland Birthing Beautiful  Communities United Way of Greater Cleveland. |  |  |  | https://cityofcleveland.legistar.com/View.ash |
| OH | Columbus, OH | 5/26/2020 | 6/2/2020 | Introduced by councilwoman and approved by city council | Councilwoman Priscilla Tyson | Y |  | N |  | Y |  | Y | City's Black people experience higher infant mortality | N |  | Provide historical context Names specific organizations to support  Emphasizes improving quality of life and health | systemic racism  criminal justice system and prison industrial complex COVID-19  SDOH  racial disparity police reform health outcomes | Recommendations for police reform Support/ funding for local organizations  Support ongoing efforts for economic development | Y | Does not give any specific | Columbus Women's Commission  My Brothers Keeper Mayors Office of Education Commission on Black Girls |  |  |  | https://[www.columbus.gov/](http://www.columbus.gov/) racismresolution/ |
| OH | Cuyahoga County | 6/23/2020 | 7/7/2020 | n/a | n/a | y |  | y |  | Y |  | y | Provides statistics on childhood asthma, incarceration, infant mortality | N | n/a | Identify known disparities and inequities  Outline policies for social/health reform | systemic racism  criminal justice system and prison industrial complex COVID-19  SDOH  racial disparities health outcomes | Status report on:  Any needed changes to county code Recommendations on employment and recruitment in county  Recommendation for data collection for racial health disparities  Improve 2012 Equity Plan for county Align goals with biennale budget | Y | Just mentions allocating funds does not specify how |  | Outlines specific actions and policies to review to help mitigate racism | <http://council.cuyahogacounty.us/> |  | <http://council.cuyahogacounty.us/pdf_counci> |
| OH | Dayton, OH | 6/17/2020 | 6/17/2020 | n/a | Ms Whaley | Y |  | N |  | Y |  | Y | County statistics on infant mortality | N |  | Identify historical disparities and inequities  Call to action to address racism | systemic racism  criminal justice system and prison industrial complex COVID-19  SDOH  racial disparities health outcomes | Support equitable policies  Adopt anti-racist lens for new policies  Partner with entities to create plan of action to address negative impacts of racism | N |  | N/A | N/A |  |  | https://[www.daytonohio.gov/DocumentCent](http://www.daytonohio.gov/DocumentCent) |
| OH | Elyria, OH | n/a | 6/24/2020 | n/a | N/A |  |  |  |  |  |  |  |  |  |  |  |  |  |  |  |  | Cannot find actual legislation just meeting minutes which do not contain any useful information |  |  | Only Could Access Meeting Notice:  https://[www.cityofelyria.org/wp-content/](http://www.cityofelyria.org/wp-content/) uploads/2020/06/  Special_Council_Notice_Wednesday_June_2 4 2020A.pdf |
| OH | Franklin County, OH | n/a | 5/19/2020 | Passed by Board of Health 5/12 | N/A | Y |  | Y |  | N |  | Y | Cities that Black residents have lower birth weights and higher infant mortality | N |  | Describes systemic racism Identifies known racial disparities  Outlines action items for board of commission to take | racial equity COVID-19  health disparities racial disparities  criminal justice system and prison industrial complex | Support/advocate for equitable policies Support community efforts  Encourage racial equity training | N |  | National Association of Counties  Franklin County Rise Together Blueprint Innovating New Pathways to Shared Prosperity Economic Inclusion Task Force |  |  |  | https://crms.franklincountyohio.gov/ RMSWeb/pdfs/  68145.FINAL_Resolution_FCPH_DeclaredRac ismPublicHealthCrisis.pdf |
| OH | Hamilton County, OH | n/a | 7/17/2020 | Draft submitted 06/24/2020 | Commissioner Victoria Parks | Y |  | Y |  | N |  | Y | Cities that Black residents have lower birth weights and higher infant mortality | N |  | Describes systemic racism Identifies known racial disparities  Outlines action items for board of commission to take | racial equity COVID-19  health disparities SDOH  healthcare access | Support local organizations/partnerships to promote health equity  Identify where racism effects infant/child mortality Address oral healthcare disparities  Improve Ohio Medicaid efforts Promote racial equity policies Conduct economic disparity study Reform police policies and training  Partner with local museum to create curriculum to educate on historical/systemic racism | N |  | National Underground Railroad Freedom Center Ohio Community Collaborative  Cradle Cincinnati  Hamilton County Oral Health Coalition  Center for Closing the Health Gap  All-In Cincinnati Gen-H | (unrelated to legislation) cannot find the actual piece of legislation only the draft | https://www.hamiltoncountyohio. |  | https://[www.hamiltoncountyohio.gov/UserFil](http://www.hamiltoncountyohio.gov/UserFil) |
|  |  |  |  |  |  |  |  |  |  |  |  |  |  |  |  |  |  |  |  |  |  |  |  |  |  |
| OH | Lima, OH | n/a | 6/22/2020 | n/a | N/A | N |  | N |  | Y |  | N |  | N |  | Provides historical context to racism and its current impact  Defines public health | redlining  COVID-19  systemic racism racial disparities health equity | Commits to open discussion on race and its impact | N |  | NAACP Lima Branch  Lima Area Black Ministerial Alliance  Allen County Public Health Western Ohio Community of Action Partnership  Lima African American Chamber of Commerce United Way of Allen County Crime Victim Services  Lima City Schools Lima Memorial Health System  Mercy Health St. Rita Medical Center  Health Partners of Western Ohio | N/A |  |  | Only Could Access Agenda:  https://[www.cityhall.lima.oh.us/](http://www.cityhall.lima.oh.us/) DocumentCenter/View/5966/City-Council- June-22-2020-Meeting-Agenda--Docket |

| OH | Lorain County, OH | n/a | 6/17/2020 | n/a | N/A | N |  | N |  | Y |  | N |  | N |  | Provides historical context to racism and its current impact  Defines public health  List policies and initials to partner/support | systemic racism racial disparities  health equity | Participate in Elyria/Lorain YWCA working group to promote diverse community  Strengthen government/community partnerships Advocate equitable practices  Call to action for Ohio state government | N |  | Elyria/Lorain YWCA |  |  |  | Only Could Access News Article:  https://chroniclet.com/news/215913/lorain- county-commissioners-declare-racism-a- public-health-crisis/? fbclid=IwAR2sm1Z19i7wyA-GEMKQx6Lc-  EiU_4KLgQ7vyxG4s6IbaPi-IbGZ6tuzYFg |
| --- | --- | --- | --- | --- | --- | --- | --- | --- | --- | --- | --- | --- | --- | --- | --- | --- | --- | --- | --- | --- | --- | --- | --- | --- | --- |
| OH | Montgomery County |  | 6/16/2020 | Similar legislation (Health Equity in all Policies) passed December 4 2019 by county Board of Health  Mrs. Lieberman moved to adopt resolution | N/A | N |  | N |  | N |  | Y | Commit resources to improving infant and maternal mortality | N |  | Defines racism and structural racism and its impact  Outlines action items | systemic racism health equity | Develop career and innovation center Expand/uphold the minority own business grant Commit to equitable policies specifically in housing, food access, job access and health well-being Strengthening community partnerships and efforts Receive regular reports on legislation's commitments | N |  | Male leadership Academy County Commissioner Association of Ohio National Association of Counties | The policy says commit to addressing things like housing but does not specify how to do so | https://onbase.mcohio.org/aspweb |  | https://[www.phdmc.org/program-](http://www.phdmc.org/program-) documents/healthy-lifestyles/gumc/ features/1812-phdmc-health-and-equity-  heiap-resolution/file |
|  |  |  |  |  |  |  |  |  |  |  |  |  |  |  |  |  |  |  |  |  |  |  |  |  |  |
| OH | Piqua, OH | n/a | 7/7/2020 | n/a | n/A | N |  | N |  | N |  | Y | Mentions racism effects infant mortality (nothing more) | N |  | Defines racism and structural racism and its impact  Outlines action items | systemic racism health equity  SDOH | Re-establish community diversity committee to look socio-economic and health effects of racism Strengthen community relationships  Support efforts to mitigate racism  Encourage state/federal government to establish | N |  |  |  |  |  | https://piquaoh.org/download/Agendas%20- |
| OH | South Euclid |  | 6/8/2020 | n/a | Joseph Frank | Y |  | N |  | Y |  | N |  | N |  | References impact of police violence References impact of COVID 19 on  Black people | COVID-19  police violence police reform | Remove discriminatory laws (i.e. Stop and Identify Statute  Expand mental health coverage regarding race Implement minimal financial penalties for false racial profiling calls to police  Expand Employment Equity Training  Reform police policies (body camera mandate, annual accountability analysis of police departments, decertification of police who engage in misconduct) | N |  |  | This was mainly focused on police reform |  |  | https://[www.cityofsoutheuclid.com/wp-](http://www.cityofsoutheuclid.com/wp-) content/uploads/2020/06/SE-06-08-20-  Council-Agenda-Legislation.pdf |
| OH | Summit County, OH | 6/1/2020 | 6/15/2020 | Take immediate effect after 8 affirmative votes | N/A | Y |  | Y |  | Y |  | Y | Cities that Black residents have lower birth weights and higher  infant mortality | N |  | Describes systemic racism Identifies known racial disparities Outlines action items for board of  commission to take | Systemic Racism COVID-19  SDOH  racial disparities health outcomes | Establish a special review committee to better understand effects of racism locally (report by 12/31/2020)  Strengthen local partnership  Support equitable employment policies Advocate/Draft polices to improve adverse childhood experiences | N |  |  |  | https://council.summitoh.net/files- |  | https://council.summitoh.net/files-legislation |
| OH | Stow, OH |  | 7/3/2020 |  | Council member Christina Shaw | Y |  | Y |  | N |  | Y | Higher Black infant mortality | N |  | Describes systemic racism Identifies known racial disparities Outlines action items for board of  commission to take | systemic racism health equity  SDOH | Supports establishment of commission for racial equity  Seek solutions for racial justice Build community relations  Promote racially equitable hiring and promotions  Advocate and draft relevant health policies | N |  |  |  |  |  | Could Only Access News Article:  https://[www.beaconjournal.com/story/](http://www.beaconjournal.com/story/) news/2020/07/04/stow-council-passes-  resolution-against-racism/42177807/ |
| OH | Upper Arlington, OH |  | 6/22/2020 | n/a | n/a | n |  | n |  | n |  | n |  | n |  | Describes racism/systemic racism and its impact  Outlines action items | systemic racism health equity  police reform | Strengthen efforts to build relationship between police and minorities  Direct Mayor to do an annual diversity and equity report  Implement community relation committee | N |  |  |  |  |  | https://docs.uaoh.net/AgendaOnline/Docume |
| OH | Westerville, OH |  | 6/16/2020 | N/a | n/a | Y | Home to several underground railroads Composition of Darling Nelly Gray in 1856 Otterbein University: one of the first universities to accept people of color Leadership Westerville's MLK project  Community Culture Day Hands across Westerville | Y |  | Y |  | Y | Mentions black infants have higher mortality rate  Black mothers have higher  mortality and morbidity | N |  | Provides historical context to racism and its current impact  Outlines action items | systemic racism health equity  racial equity | Seek solutions for racial justice/equity Build/strengthen community relations  Promote racially equitable hiring and promotions Review city policies/systems with racial equity lens  Advocate and draft relevant health policies | N |  | Franklin County Public Health, Westerville City Schools, the Westerville Public Library, the Westerville Area Chamber of Commerce, and Otterbein University and its Truth, Racial Healing, and Transformation Campus  Center |  |  |  | https://[www.morpc.org/wordpress/wp-conte](http://www.morpc.org/wordpress/wp-conte) |
| OH | Warren, OH | n/a | 6/24/2020 | n/a | n/a | N |  | N |  | Y |  | Y | Racism leads to higher infant mortality | N |  | Provides historical context to racism and its current impact | Racial equity Health equity | Commits to improving minority quality of life  (specifically poverty and economic mobility) through a systemic data driven focus | N |  |  |  | https://[www.warren.org/images/P](http://www.warren.org/images/P) | D | https://[www.warren.org/images/PDFs/city-](http://www.warren.org/images/PDFs/city-) council/minutes-legislation/2020/06-24-  20_RES_4660-2020.pdf |

| OH | Youngstown, OH | 6/19/2020 | 6/23/2020 | n/a | n/a | Y |  | N |  | N |  | Y | Black infant mortality is the county is one of the highest in the nation | N |  | Describes racism (systemic and individual)  Identifies known racial disparities | Racial equity health outcomes  systemic racism COVID 19 | Commits to improving quality of life for racial minorities | N |  | Youngstown City Health District  Mahonging county public health |  | https://[www.wfmj.com/story/4227](http://www.wfmj.com/story/4227) | https://youngstownohio.gov/sites/ | Only Could Access Meeting Minutes:  https://youngstownohio.gov/sites/default/ files/council_minutes/September%2022%2C  %202020/Minutes%20June%2019%2C  %202020.pdf |
| --- | --- | --- | --- | --- | --- | --- | --- | --- | --- | --- | --- | --- | --- | --- | --- | --- | --- | --- | --- | --- | --- | --- | --- | --- | --- |
| OK | Ardmore, OK | n/a | 6/5/2020 | n/a | n/a | N |  | N |  | Y |  | N |  | N |  | Denounces racism | racial disparities | Consider tools to eliminate racism | N |  |  |  |  |  | https://docs.google.com/viewerng/viewer? url=https://[www.ardmorecity.org/](http://www.ardmorecity.org/) AgendaCenter/ViewFile/Item/6028?fileID  %3D3823 |
| PA | Allegheny County, PA | n/a | 5/5/2020 | motion presented during county council meeting | Council Members Bennett and Walton | Y |  | N |  | N |  | Y | Cities that Black residents have higher infant mortality to comparable cities and higher  infant mortality | N |  | Defines racism and structural racism and its impact  References local statistics on MCH and life expectancy  Outlines action items | racial equity Health disparities systemic racism  criminal justice system and prison industrial complex | Create internal policies, procedures, and assessments for racial equity  Incorporate policies that promote/educate about racial equity and diversity in organizational structures  Advocate for relevant policies that improve health in communities of color (i.e. Black Mamas Matter Agenda)  Build/strengthen ties with governmental organization that address racism | N |  | Black Mamas Matter University of Pittsburgh |  |  |  | https://alleghenycounty.legistar.com/Legislati |
| PA | Erie, PA |  | 9/8/2020 | motion by Carl Anderson III seconded Kimberly Clear  passed 5 to 2 motion | n/a | Y | Breaks down statistics by health, housing,  jobs/economics, education | N |  | Y |  | Y | Black people have lower birth weight  Black people receive less prenatal care  Higher infant mortality rate | N |  | Defines racism and structural racism and its impact  References local statistic housing, health, job/economics, education  Outlines action items | racial equity health disparities health outcome systemic racism  criminal justice system and  prison industrial complex | Commits to addressing racism  Review and recommend departmental changes to eliminate/reduce disparities with a status report | N |  |  |  |  |  | <http://public.eriecountypa.gov/councildocs/> resolutions/2020-43.pdf |
| PA | Pittsburgh, PA | 10/22/2019 | 12/17/2019 | presented to council 10/22/2019 recommended by committee 12/13/2019 amended and passed 12/11/2019  signed by mayor 12/23/2019 | Reverend Ricky V. Burgess,  R. Daniel Lavelle, Bruce A. Kraus | Y |  | N |  | N |  | y | Higher Black infant mortality | N |  | Defines racism and structural racism and its impact  Outlines action items | racial equity  criminal justice system and prison industrial complex health disparities  systemic racism | Create internal policies, procedures, and assessments for racial equity  Incorporate policies that promote/educate about racial equity and diversity in organizational structures  Advocate for relevant policies that improve health in communities of color (i.e. Black Mamas Matter Agenda)  Build/strengthen ties with governmental organization that address racism | N |  | Black Mamas Matter |  |  |  | https://pittsburgh.legistar.com/LegislationDet |
| TN | Chattanooga | 7/7/2020 | 7/7/2020 | Called for by Coonrod on June 30th; Adopted 6- 2 | Councilwoman Coonrod | N |  | N |  | Y |  | Y | higher rates of infant mortality in African Americans as a repercussion of historical racism; also mentions bias in the medical  professions towards black women | N |  | Define racism and its history Examples of Racism (COVID, Prison Industrial complex, etc.)  The city and community's role | COVID-19  Black Lives Matter police violence police reform redlining  criminal justice system and prison industrial complex healthcare access transparency to community SDOH  systemic racism  interpersonal racism | forming the Mayor’s Office of Community Resilience;  urge our community to immediately take steps to address, fund, and support areas to strategically  reduce the long-term impact of SDOH; | Y | Urge community to fund areas to reduce impact of SDOH |  |  | <http://www.chattanooga.gov/city-> council-files/Agenda-Minutes/  Minutes/2020/07_07_2020.pdf | https://newschannel9.com/news/l | <http://www.chattanooga.gov/city-council-file> |
| TN | Memphis | 7/7/2020 | 7/7/2020 | adopted and approved by City Council regular session | Chairwoman Patrice Robinson | Y | Cites the violence to Black Americans during the May 1 to May 3 Memphis  Massacre of 1866 | N |  | Y |  | N |  | N |  | Cites the county declaration; History of racism in the city and US; present consequences of racism for people of  color; actions/commitments | systemic racism COVID-19  healthcare access health equity  police violence | Engage with the Shelby County board of commissioners to address racism and combat its impacts; work with partners to address health  inequities for people of color | N |  |  |  | https://docs.google.com/gview? url=https%3A%2F  %2Fmemphis.granicus.com  %2FDocumentViewer.php%3Ffile  %3Dmemphis_c590e1b41f930b0d 7f40821c9ad80663.pdf%26view  %3D1&embedded=true |  | https://docs.google.com/gview?url=https%3A |
| TN | Shelby County | 6/17/2020 | 6/22/2020 | On agenda 6/17/2020, approved 10-2 on 6/22 | County Commissioner Tami Sawyer | N |  | N |  | N |  | N |  | N |  | Declares Racism a pandemic | systemic racism COVID-19 | enact policies that defend minorities and eradicate systemic racism | N |  |  | Refers to racism as a "pandemic" | https://[www.shelbycountytn.gov/D](http://www.shelbycountytn.gov/D) |  | Only able to access agenda:  https://[www.shelbycountytn.gov/](http://www.shelbycountytn.gov/) DocumentCenter/View/36948/REVISED- Committee-Meeting-Agenda-for-June-17-  2020 |
| TX | Austin | 7/29/2020 | 7/29/2020 | passed unanimously 11-0 | Council Member Natasha Harper-Madison | Y | Mentions 1928 Master Plan that acted as legalized form  of segregation | N |  | N |  | Y | Higher Levels of lower birth weights | N |  | Defining Racism and the history of racism in the City; Impacts of racism on communities of color with city specific statistics; the cities past work;  action steps | interpersonal racism systemic racism SDOH  racial disparities COVID-19  task force health equity  health outcomes | Review recommendations from Mayor's Task Force in areas related to Education, real estate/housing, health, Finances/Banking, and Civil & Criminal Justice; advocate locally and through the National League of Cities and Texas Municipal League for relevant policies to improve health of communities  of color | N |  |  |  | https://[www.kxan.com/racial-](http://www.kxan.com/racial-) justice-and-equality-movement/ austin-council-declares-racism-a- public-health-crisis-thats-killing-  black-and-brown-people/ |  | https://[www.austintexas.gov/edims/](http://www.austintexas.gov/edims/) document.cfm?id=344245 |
| TX | Dallas County | 6/16/2020 | 6/16/2020 | Unanimously approved by 5 member body | Commissioner John Wiley Price | N |  | Y | Disproportionality in incarceration rates in Texas Prison System for Black  people | N |  | Y | Black residents also have higher levels of infant mortality and  lower birth weights | N |  | County's past efforts to address racism; defining racism along with consequences on people of color;  Action steps | criminal justice system and prison industrial complex interpersonal racism systemic Racism  SDOH  racial disparities health outcomes health equity COVID-19  Black Lives Matter | enhance diversity to progress equity and justice oriented organization; promote equity through all policies to dismantle systemic racism; advocate locally for relevant policies; solidify alliances and partnerships with other organisms fighting racism; support community efforts to amplify issues of racism; promote and encourage racial equity  training; identify clear goals and periodic reports | N |  |  |  |  |  | https://dallascounty.civicweb.net/document/ |
| TX | Harris County | 6/30/2020 | 6/30/2020 | Presented by commissioner Ellis, approved by 3-2 vote | presented by Commissioner Rodney Ellis | Y | Mentions that County Attorney's office used to be  a slave auction site | Y | Texas Rangers' oppression/ murder of Black and  Indigenous communities | Y | Homestead Act, Black Codes, Wagner Act and Fair Labor Standards Act, Home Owners Loan Corporation maps, War on  Drugs | Y | Black mothers and infants die at twice the rate of their White  counterparts | N |  | Overview of historical racism at the city, county, state, and national level Statistics of health and other racial disparities of black residents in the county  Action Steps | COVID-19  health outcomes redlining  criminal justice system and prison industrial complex systemic racism interpersonal racism centering community experiences and voices healthcare access  health equity | develop policies, programs, services to dismantle systemic racism;  develop partnerships with community impacted by racism;  ensure complete and regular availability of specific race and ethnicity data;  commits to ongoing work around race and equity | N |  |  | there is a plan in place to amend to include gender (i.e. racism and gender as a public  health crisis) | https://thetexan.news/harris-count | https://[www.houstontx.gov/health](http://www.houstontx.gov/health) | https://thetexan.news/harris-county-declares |

| TX | San Antonio | 8/20/2020 | 8/20/2020 | 9 affirmative, 1 abstention (9-0-1) | Councilwoman Jada Andrews-Sullivan and Councilwoman Ana Sandoval | Y | Redlining, segregated lunch counters at Woolworth's | Y | Mentions the 1836 constitution when Anglo Slaveholders seceded from Mexico and Mexicans experienced mob violence and murder; also mentions Texas Rangers and their violence and murder  towards people of color | Y | Emphasis on Latinx Community | Y | the rate of infant mortality among Black people in the county is almost 50% higher than those that are Latinx or white; Low  birthrates almost 60% higher | N |  | The city's past relevant work in this area of racial equity; Defining racism along with historical and present consequences for people of Color in the state; County and City stats related to economic and educational disparities; response of national and international health organizations ;  Action Steps | center Community experiences and voices  health outcomes health equity interpersonal racism systemic racism redlining  racial justice SDOH  healthcare access | Advocate to include state and federal legislation priorities; support community based groups; City staff will present bi-annual presentation updating community on policies and programs; advance racial equity through policies, engagement of marginalized communities, improvement of data systems, health equity, city services, and mitigating  housing and job displacement | N |  | APHA AMA  American Academy of Pediatrics  American College of Emergency Physicians WHO  American College of Physicians |  | https://[www.sanantonio.gov/gpa/](http://www.sanantonio.gov/gpa/) News/ArtMID/24373/ArticleID/ 19335/City-Council-Officially- Declares-Racism-a-Public-Health-  Crisis |  | https://[www.apha.org/topics-and-issues/heal](http://www.apha.org/topics-and-issues/heal) |
| --- | --- | --- | --- | --- | --- | --- | --- | --- | --- | --- | --- | --- | --- | --- | --- | --- | --- | --- | --- | --- | --- | --- | --- | --- | --- |
| VT | Burlington | 7/16/2020 | 7/16/2020 | community declaration by Mayor, Vermont Racial Justice Alliance, and 30+ County Organizations | Mayor Miro Weinberger | N |  | N |  | N |  | N |  | N |  | Defining racism and its impact on black residents in the city and county; Action steps/commitments; List of 30+  organizations involved in this declaration | health equity systemic racism health outcomes COVID-19  SDOH | take a coordinated effort working with great collaboration and urgency;  commit to eradicating systemic racism as organizations, actively fighting racist practices and participating in the creation of more just and equitable systems;  coordinate work and participate in ongoing joint action, grounded in science and data | N |  | 30+ County Organizations |  | https://[www.burlingtonvt.gov/](http://www.burlingtonvt.gov/) Press/mayor-miro-weinberger-  vermont-racial-justice-alliance- and-30-plus-chittenden-county |  | https://webpubcontent.gray.tv/wcax/docs/20 |
| WA | King County | 6/11/2020 | 6/18/2020 | Introduced and passed (amended by Board of Health) 13 yes, 1 excused | King County Executive Dow Constantine and Public Health — Seattle & King County Director Patty  Hayes | Y |  | N |  | Y |  | Y | data shows how BIPOC communities are affected by maternal and infant mortality, as  well as underweight babies | N |  | Defining Racism; Impact of racism on communities of color; past work done by other relevant organizations and  county; Action steps | systemic racism police violence  criminal justice system and prison industrial complex COVID-19  centering community experiences and voices | commitment to partnership with community organizations and leaders ; using qualitative data about racial inequities; assessing and writing policies with racial justice lens; working around race  equity and participation in training by Board members | N |  |  | Joint declaration with county and county board of health | <http://mrsc.org/getmedia/cfd050d> | https://[www.kingcounty.gov/elect](http://www.kingcounty.gov/elect) | <http://mrsc.org/getmedia/cfd050db-4a7a-491> |
| WI | Cudahy | 6/23/2020 | 6/23/2020 | Common Council Passed 3-1 | Alderman Justin Moralez | N |  | N |  | N |  | Y | The state infant mortality rate of  non-Hispanic black women is the highest in the nation | N |  | Defining Racism; Impact of racism on communities of color; work done by  other relevant organizations; Action steps | interpersonal Racism systemic racism  criminal justice system and prison industrial complex SDOH  racial justice COVID-19 | Conduct an assessment of internal policy and procedures to ensure racial equity; create an equity and justice oriented organization; incorporate educational efforts to dismantle racism in the organizational work plan; advocate  for relevant policies to improve health in communities of color | Y | City Common Council considering the City's Budget for  allocating adequate financial resources for action steps | APHA  Wisconsin Public Health Association |  | https://[www.cudahy-wi.gov/CC%2](http://www.cudahy-wi.gov/CC%252) | 0 | https://[www.cudahy-wi.gov/CC%20PACKET%2](http://www.cudahy-wi.gov/CC%20PACKET%252) |
| WI | Dane County | 6/15/2020 | 7/13/2020 | Referred from county board to personnel and finance committee (6/19/20); personnel and finance committee recommend for approval (7/6/20); Executive Committee recommended  for approval (7/9/20); county board adopted 7/9/20 and county executive signed 7/13 | moved by County Board  Supervisor Shelia Stubbs, District 23 | N |  | N |  | N |  | Y | In the state, the infant mortality rate for infants of non-Hispanic  black women is the highest in the nation | N |  | Defines Racism and its consequences (specifically in the State);  Shows the past and current efforts by state and county regarding racism and health disparities;  Action Steps | systemic racism interpersonal racism SDOH  criminal justice system and  prison industrial complex health outcomes | continue to use racial equity and social justice lens in approaching policies to dismantle systemic  racism; commits to past, current, and future efforts to dismantle racism; | N |  | Criminal Justice Council (local)  Tamara D. Grigsby Office for Equity and Inclusion (local) |  | https://dane.legistar.com/Legislatio |  | https://dane.legistar.com/LegislationDetail.as |
| WI | Kenosha County | 8/5/2020 | 8/5/2020 | approved 18-1 | Supervisor Laura Belsky | N |  | N |  | Y |  | Y | The infant mortality rate of non- Hispanic black infants is the highest in the nation; black mothers and babies in the county experience higher infant mortality, inadequate prenatal care, low birth weight, lower rates of breastfeeding upon hospital  discharge | N |  | Defining racism along with its impacts on black residents; County specific statistics on health economic and  education disparities; action steps | health Outcomes SDOH  systemic racism interpersonal racism criminal justice system and prison industrial complex redlining  racial justice | Assessing internal policy and procedures to ensure racial equity;  create an inclusive organization with increased diversity;  Have inclusion and equity and organizational practice and education;  Advocate for relevant policies to improve health in communities of color;  Encourage other local, state, and national entities to recognize racism as a public health crisis | N |  |  |  |  |  | https://[www.kenoshacounty.org/AgendaCent](http://www.kenoshacounty.org/AgendaCent) |
| WI | Milwaukee (city) | 6/18/2019 | 7/30/2019 | Adopted 13 to 0 after referral to Steering and Rules Committee | Common Council | N |  | N |  | N |  | Y | average infant mortality rate in the City of Milwaukee among African-American mothers was  15.4 deaths per 1000 births (vs  5.1 for white mothers) | N |  | Impact of racism in the city; The cities past response and actions to racism; Commitments/Action steps (going  forward) | centering community experiences and voices health equity  SDOH  interpersonal racism systemic racism  criminal justice system and prison industrial complex racial justice  health outcomes | Organizational Infrastructure (processes to improve racial equity, health outcomes, and SDOH) Organization and Workforce Capacity (policies related to racial equity in recruitment, hiring, training, education, organizational structure) Internal Practices and Processes (policies to address health equity, track resource allocation) Policy and Legislative Change (advocate for policies related to health equity)  Community Alliance Building | Y | Identify and track resource allocation | Equal Rights Commission (Milwaukee) Government Alliance for  Racial Equity (GARE) |  | https://milwaukee.legistar.com/Leg |  | https://milwaukee.legistar.com/LegislationDe |
| WI | Milwaukee County | 4/8/2019 | 5/20/2019 | Health and Human Needs Committee recommend for adoption on 4/17/19; Board of Supervisors adopt 4/25/19; County Executive  signed 5/20/19 | County Executive Abele and Supervisory Nicholson | N |  | N |  | N |  | Y | The state infant mortality rate of non-Hispanic black women is the highest in the nation; 2016 Infant mortality rate in the County for African American mothers was  13.1 deaths per 1,000 births (as compared to 4.9 per 1,000 for white mothers); lower rates of  prenatal care for African American mothers as well | N |  | Defining Racism; Impact of racism on communities of color; work done by other relevant organizations; Action  steps | police violence racial disparities health outcomes SDOH  systemic racism interpersonal racism criminal justice system and prison industrial complex health equity  racial justice | Assess internal policy and procedures to ensure racial equity; work to create inclusive organization identifying specific activities to increase diversity as well as being equitable and providing education and training; advocate for relevant policies that improve health in communities of color; support  and encourage local, state, and federal initiatives and declarations | N |  | APHA |  | https://milwaukeecounty.legistar.c |  | https://milwaukeecounty.legistar.com/Legisla |
| WI | Rock County | 6/25/2020 | 6/25/2020 | Unanimously approved by acclamation | Supervisors Peer and Rashkin | N |  | N |  | N |  | N |  | N |  | Recommendations and commitments | systemic racism health outcomes | Advocate for polices to improve health of communities of color; eliminate health and opportunity gaps along racial lines; support local, state, and federal initiatives to advance social justice; advocacy to dismantle systemic racism; supervisors will work to build alliances and  partnerships with other organizations confronting racism | N |  |  |  | https://[www.co.rock.wi.us/compon](http://www.co.rock.wi.us/compon) |  | https://[www.co.rock.wi.us/component/easyfo](http://www.co.rock.wi.us/component/easyfo) |
| WI | State at Large | n/a | 6/4/2020 | Statement made during the governors  Department of Health Services weekly COVID- 19 briefing | Governor Tony Evers | N |  | N |  | N |  | N |  | N |  | verbally stating racism as a public health crisis; stating statistics of racial disparities in the state; acknowledges  police violence in the US | police violence  criminal justice system and prison industrial complex racial disparities  systemic racism police reform | call for legislature to pass a bill restricting use of force techniques; | N |  |  |  |  |  |  |
|  |  |  |  |  |  |  |  |  |  |  |  |  |  |  |  |  | **Frequency count of themes** |  |  |  |  |  |  |  |  |
|  |  |  |  |  |  |  |  |  |  |  |  |  |  |  |  |  |  |  |  |  |  |  |  |  |  |
|  |  |  | 0 |  |  | 38 | Yes, included | 18 | Yes, included | 58 | Yes, included | 66 | Yes, mentioned | 3 | Yes, mentioned | *Black Lives Matter* | 12 |  | 21 | Yes, discussed |  |  |  |  |  |
|  |  |  |  |  |  |  |  |  |  |  |  |  |  |  |  | **Health Outcomes*** | 63 |  |  |  |  |  |  |  |  |
|  |  |  |  |  |  |  |  |  |  |  |  |  |  |  |  | **Social Determinants of Health (SDOH)** | 66 | 0 |  |  |  |  |  |  |  |
|  |  |  |  |  |  |  |  |  |  |  |  |  |  |  |  | Police violence | 28 |  |  |  |  |  |  |  |  |
|  |  |  |  |  |  |  |  |  |  |  |  |  |  |  |  | Redlining | 19 |  |  |  |  |  |  |  |  |
|  |  |  |  |  |  |  |  |  |  |  |  |  |  |  |  | **Covid-19** | 76 |  |  |  |  |  |  |  |  |
|  |  |  |  |  |  |  |  |  |  |  |  |  |  |  |  | Health equity | 44 |  |  |  |  |  |  |  |  |
|  |  |  |  |  |  |  |  |  |  |  |  |  |  |  |  | Interpersonal racism | 45 |  |  |  |  |  |  |  |  |
|  |  |  |  |  |  |  |  |  |  |  |  |  |  |  |  | **Systemic racism** | 92 |  |  |  |  |  |  |  |  |
|  |  |  |  |  |  |  |  |  |  |  |  |  |  |  |  | racial disparities (not health specific, e.g. job loss) | 41 |  |  |  |  |  |  |  |  |
|  |  |  |  |  |  |  |  |  |  |  |  |  |  |  |  | centering community experiences and voices | 25 |  |  |  |  |  |  |  |  |
|  |  |  |  |  |  |  |  |  |  |  |  |  |  |  |  | *hate crimes* | 5 |  |  |  |  |  |  |  |  |
|  |  |  |  |  |  |  |  |  |  |  |  |  |  |  |  | racial justice | 26 |  |  |  |  |  |  |  |  |
|  |  |  |  |  |  |  |  |  |  |  |  |  |  |  |  | police reform | 13 |  |  |  |  |  |  |  |  |
|  |  |  |  |  |  |  |  |  |  |  |  |  |  |  |  | *transparency to community* | 2 |  |  |  |  |  |  |  |  |
|  |  |  |  |  |  |  |  |  |  |  |  |  |  |  |  | criminal justice system and prison industrial complex | 61 | 59 |  |  |  |  |  |  |  |

|  |  |  |  |  |  |  |  |  |  |  |  |  |  |  |  | *Poverty through policies* | 0 |  |  |  |  |  |  |  |  |
| --- | --- | --- | --- | --- | --- | --- | --- | --- | --- | --- | --- | --- | --- | --- | --- | --- | --- | --- | --- | --- | --- | --- | --- | --- | --- |
|  |  |  |  |  |  |  |  |  |  |  |  |  |  |  |  | *Task Force* | 10 |  |  |  |  |  |  |  |  |
|  |  |  |  |  |  |  |  |  |  |  |  |  |  |  |  | Healthcare Access | 28 | 29 |  |  |  |  |  |  |  |
