# Supplementary material for: Racism as Public Health Crisis: Assessment and Review of Municipal Declarations and Resolutions Across the United States
Source: Front Public Health. 2021 Aug 11;9:686807. doi: 10.3389/fpubh.2021.686807 (PMC8385329; doi:10.3389/fpubh.2021.686807)
Supplement: Supplementary file 2 [file Table_2.DOCX]

**Supplementary Table 1B. Summary of common language across multiple declarations and resolutions**

| **State(s)** | **Example Text 1** | **Municipalities including this text** | **Example Text 2** | **Municipalities including this text** |
| --- | --- | --- | --- | --- |
| **Connecticut** | Nearly Identical 8-10 Point Action Section: “identify clear goals and objectives, including periodic reports... to assess progress and capitalize on opportunities to further advance racial equity” | Bloomfield, Bridgeport, Hamden, Manchester, New Britain, New Haven, New London, Simsbury, West Hartford, Windham, Windsor | “racism as a root cause of poverty and constricted economic mobility” | Bloomfield, Bridgeport, Easton, Hamden, Manchester, New Britain, New Haven, New London, Simsbury, West Hartford, Windham, Windsor |
| **Massachusetts** | “racial justice is the creation and proactive reinforcement of policies, practices, attitudes, and actions that produce equitable power, access, opportunity, treatment, and outcomes for all people regardless of race” | Beverly, Boston, Framingham | “Focus on access to prevention and treatment that is culturally and linguistically competent and meets communities where they are to counter the inequities that exist in health care” | Beverly, Boston, Framingham |
| **Ohio** | Nearly identical: “almost all of the 400 years of black America’s experience was under slavery and Jim Crow laws which allowed preferential opportunity to some while at the same time subjected people of color to hardship and disadvantage in all areas of life” | Athens, Akron, Cleveland, Cuyahoga County, Dayton, Lima, Summit County | Nearly identical: “racism acts on systemic, institutional and interpersonal levels, all of which operate throughout time and across generations” | Akron, Cleveland, Dayton, Lima, Summit County |
| **Wisconsin** | “responsibility to address racism, including seeking solutions to reshape the discourse and actively engaging all citizens in racial justice work” | Cudahy, Kenosha County, Milwaukee City, Milwaukee County | “Healthiest Wisconsin 2020 asserts that, ‘Wisconsin must address persistent disparities in health outcomes, and the social, economic, educational and environmental inequities that contribute to them’ ” | Cudahy, Dane County, Kenosha County, Milwaukee City, Milwaukee County |
| **California, Connecticut, Florida, Georgia, Michigan, Texas** | Nearly Identical 8-10 Point Action Section: “identify clear goals and objectives, including periodic reports... to assess progress and capitalize on opportunities to further advance racial equity” | Los Angeles, Bloomfield, Hillsborough County, DeKalb County, Ypsilanti, Dallas | “racism as a root cause of poverty and constricted economic mobility” | Los Angeles, Bloomfield, Hillsborough County, DeKalb County, Dallas |
