# Supplementary material for: Racism as Public Health Crisis: Assessment and Review of Municipal Declarations and Resolutions Across the United States
Source: Front Public Health. 2021 Aug 11;9:686807. doi: 10.3389/fpubh.2021.686807 (PMC8385329; doi:10.3389/fpubh.2021.686807)
Supplement: Supplementary file 3 [file Table_3.DOCX]

**Appendix 1C: Social Media Thread: Response to Question about Updates and Actions related to Racism as a Public Health Crisis**

| **Municipality** | **Twitter comments** | **Websites/Twitter Handles** |
| --- | --- | --- |
| Tulsa, OK | [Tulsa] has the equality indicators report and the race massacre graves location project going on. | [@cityoftulsagov](https://twitter.com/cityoftulsagov) |
| Portland, ME | Portland Maine has a new racial equity committee. | [@CityPortland](https://twitter.com/CityPortland)  [http://portlandmaine.gov/2658/Racial-Equity-Steering-Committee](https://t.co/sloALrTJOJ?amp=1) |
| South Portland, ME | South Portland started a human rights commission | <https://southportland.org/our-city/board-and-committees/human-rights-commission/> |
| Freeport, Maine | Freeport Maine is forming an equity committee: | [https://freeportmaine.com/home/events/17](https://t.co/hQzU6sFJHd?amp=1) |
| Washtenaw Co, MI | None | [https://washtenaw.org/3182/Racial-Justice-and-Health-Equity](https://t.co/gQpGvMjEAf?amp=1)  [@wcpublichealth](https://twitter.com/wcpublichealth) |
| Los Angeles, CA | Los Angeles Department of Public Health has a Center for Health Equity | [http://publichealth.lacounty.gov/centerforhealt](https://t.co/5VedukYW0H?amp=1) |
|  | There has been greater incorporation into climate resilience/equity/justice plans. I don’t know how community up these are in creation & implementation | https://www.greenbiz.com/article/8-cities-share-how-racial-justice-embedded-their-climate-plans |
| Milwaukee, WI | Milwaukee’s climate equity plan comes w policy recommendations which I think is rare. | https://city.milwaukee.gov/climate/Climate-Plan |
| Burlington, VT | Burlington established a police oversight commission and committed to reduce the number of uniformed officers by 30% by attrition -- but now the headcount cap may be revised upwards again per the commission due to early-morning staffing shortage concerns | https://vtdigger.org/2021/01/13/commission-recommends-council-raise-burlington-police-head-count/ |
| Carrboro, NC | Among other things, the Carrboro Town Council this week established a racial equity commission that will be tasked with making recommendations to address systemic | None |
|  | Middletown, CT formed a committee to work on their plan as did Windsor and Manchester | [@TDEverette](https://twitter.com/TDEverette)  Is the Exec Director of group that held webinar on next steps. |
| Washington, DC | Here in DC the council voted in favor of a resolution declaring racism a public health crisis. Bill introduced by council member  [@kenyanmcduffie](https://twitter.com/kenyanmcduffie) | See proposed resolution and committee actions here: [https://lims.dccouncil.us/downloads/LIMS](https://t.co/DBrDAePk4c?amp=1) |
|  | Passed the REACH Act: The REACH Act, The Racial Equity Achieves Results Act of 2020 | <https://kenyanmcduffieward5.com/equity/> |
